# Self‐assembled Ru(bda) Coordination Oligomers as Efficient Catalysts for Visible Light‐Driven Water Oxidation in Pure Water

**DOI:** 10.1002/anie.202211445

**Published:** 2022-11-23

**Authors:** Tim Schlossarek, Vladimir Stepanenko, Florian Beuerle, Frank Würthner

**Affiliations:** ^1^ Institut für Organische Chemie Universität Würzburg Am Hubland 97074 Würzburg Germany; ^2^ Center for Nanosystems Chemistry (CNC) Universität Würzburg Theodor-Boveri-Weg 97074 Würzburg Germany

**Keywords:** Artificial Photosynthesis, Coordination Oligomer, Photocatalysis, Ruthenium Complexes, Water Oxidation

## Abstract

Water‐soluble multinuclear complexes based on ruthenium 2,2′‐bipyridine‐6,6′‐dicarboxylate (bda) and ditopic bipyridine linker units are investigated in three‐component visible light‐driven water oxidation catalysis. Systematic studies revealed a strong enhancement of the catalytic efficiency in the absence of organic co‐solvents and with increasing oligomer length. In‐depth kinetic and morphological investigations suggest that the enhanced performance is induced by the self‐assembly of linear Ru(bda) oligomers into aggregated superstructures. The obtained turnover frequencies (up to 14.9 s^−1^) and turnover numbers (more than 1000) per ruthenium center are the highest reported so far for Ru(bda)‐based photocatalytic water oxidation systems.

## Graphical abstract

Water‐soluble Ru(bda) coordination oligomers with varying lengths are investigated in visible light‐driven water oxidation catalysis. The excellent performance and stability of longer oligomers in pure water is rationalized by the very high local concentrations of active sites in self‐assembled superstructures.

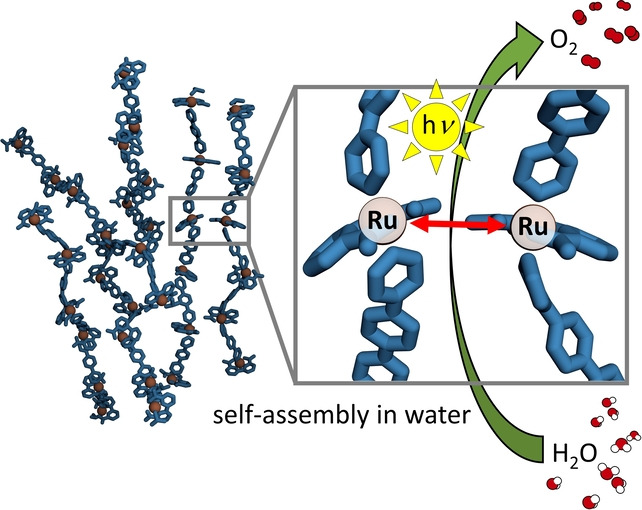

The sun emits an enormous amount of energy and its conversion into storable energy carriers will be the basis for a sustainable future.^[1]^ In this regard, artificial photosynthetic systems that split water to produce solar hydrogen have gained increasing attention.[^2^, ^3^, ^4^] The most crucial step on the way towards real applications is the optimization of the water oxidation half‐reaction. This is challenging due to the sluggish kinetics of this proton‐coupled four‐electron transfer process.^[5]^ To meet this challenge, various artificial systems have been developed and those based on ruthenium and iridium catalysts have emerged as the most promising candidates.^[6]^ The major focus lies on ruthenium complexes featuring 2,2′‐bipyridine‐6,6′‐dicarboxylate (bda) and pyridines as additional axial ligands, which have proved to be highly efficient water oxidation catalysts (WOCs).[^7^, ^8^, ^9^] Routinely, these catalysts are evaluated in chemical assays using (NH_4_)_2_Ce(NO_3_)_6_ (CAN) as the sacrificial chemical oxidant. Over the last four decades, this allowed to monitor a remarkable evolution of catalyst efficiency with an increase in turnover frequencies (TOF) from 0.002 s^−1^ in 1982^[10]^ to more than 1000 s^−1^ nowadays.^[11]^ However, highly acidic conditions (pH<1) are required, as well as a large excess of CAN (Ce^IV^/Ce^III^ *E* ^0^=+1.61 V vs NHE) as chemical fuel, thus rendering such conditions unsuitable for actual energy production.^[12]^ As a more sustainable alternative, visible light‐driven water oxidation was introduced utilizing WOCs in combination with a photosensitizer,^[13]^ e.g., Ru(bpy)_3_^2+^, and a sacrificial electron acceptor, e.g., Na_2_S_2_O_8_.[^12^, ^14^] Due to the increasing complexity of these three‐component systems, only a small number of WOCs have been studied so far under photocatalytic conditions[^15^, ^16^, ^17^, ^18^, ^19^, ^20^] and considerably lower TOFs are typically obtained due to more complex photophysics and additional deactivation pathways. To improve both performance and stability, recent studies emphasized the importance of supramolecular interactions between closely arranged molecular WOCs.[^21^, ^22^, ^23^, ^24^, ^25^, ^26^, ^27^] Therefore, we envision higher TOF and TON values can be achieved by the accumulation of individual catalysts in coordination oligomers.[^28^, ^29^] As an added benefit, such materials are typically easier to synthesize and purify than defined mono‐ or multinuclear molecular species.

However, the low solubility of the hydrophobic Ru(bda) system in pure water also hampers real‐life applications as visible‐light‐driven WOCs. Typically, organic co‐solvents, i.e., acetonitrile or trifluoroethanol, must be added to the aqueous reaction mixture to ensure the homogeneous dissolution of all necessary components.[^30^, ^31^] Therefore, following our previous research on oligo(ethylene glycol) chain functionalized Ru(bda) macrocycles,^[32]^ here we introduce oligo(ethylene glycol) chains to the central benzene moiety of a new bifunctional 1,4‐di(pyridin‐4‐yl)benzene linker **L** in linear oligomeric [Ru(bda)L]_*n*_ catalysts for visible light‐driven water oxidation in pure water. Beyond the expected improvement of the performance and stability of these systems under operating conditions, due to the lack of competitive coordination of acetonitrile solvent molecules and concomitant ligand dissociation at the ruthenium center,^[32]^ we observed an astonishing change from second to first‐order kinetics upon increasing oligomer length. According to our analyses, the concomitant improvement of catalytic performance originates from the self‐assembly of the linear Ru(bda) oligomers.

Ru(bda) oligomers **4** were prepared via multiple ligand exchange reactions between the water‐solubilizing bipyridine derivative **1** and ruthenium precursor complexes **2** or **3** in methanol (Scheme 1). Under optimized reaction conditions, long oligomers **4 a** were obtained (Figures S1, S2, see Supporting Information for more details). For comparison, shorter oligomers **4 b** were synthesized from an adjusted ratio between **1** and **2**. ^1^H NMR spectroscopy for the two different materials reveals the simultaneous presence of bipyridine and dmso end‐units for **4 a**, while **4 b** only features dmso end‐units. The oligomers were purified by three consecutive precipitations of the crude material from chloroform with *n*‐hexane resulting in high yields of 76 % and 96 % for **4 a** and **4 b**, respectively. As an additional molecular reference, dinuclear complex **4 c** was synthesized from linker **1** and [Ru(bda)(dmso)(4‐pic)] (**3**) precursor complex to compare the catalytic properties of the oligomeric materials to a smaller and defined reference complex based on similar building blocks.

**Scheme 1:**
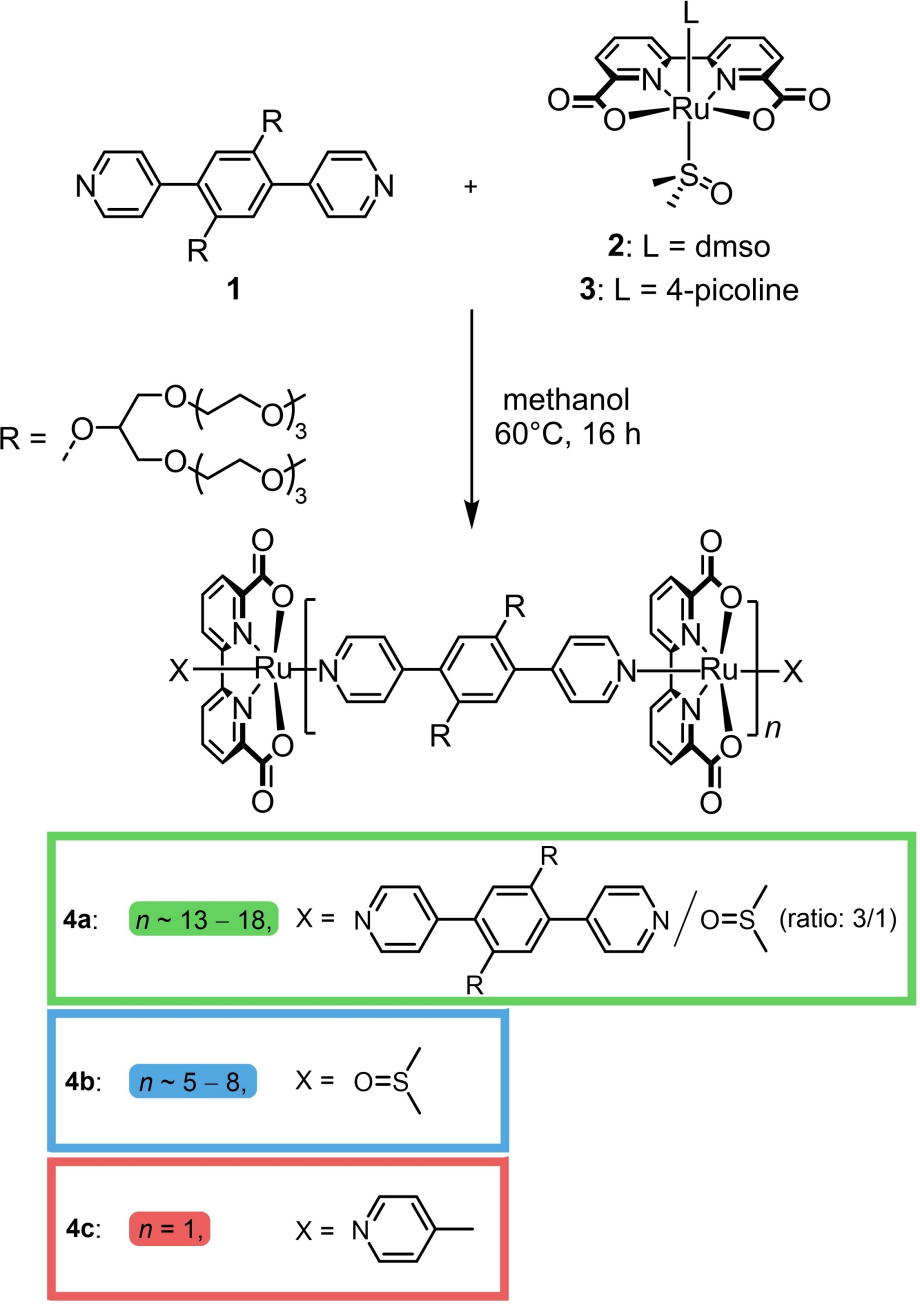
Synthesis of Ru(bda) oligomers **4 a**–**c** from ditopic linker **1** and Ru(bda)(dmso)_2_ (**2**) or Ru(bda)(dmso)(4‐pic) (**3**), respectively.

The length distribution within the two oligomeric materials, **4 a** and **4 b**, was analyzed by several complementary techniques. Initially, end‐group analysis for the ^1^H NMR spectra revealed an average number of 17–18 for **4 a** and 6–7 repeating units for **4 b** (Figure 1d, Figure S3). These size distributions were further confirmed by DOSY NMR data for the two oligomer fractions, analyzed using the log‐normal distribution model[^33^, ^34^] (Figure 1c, bottom, Figure S9–S11, Table S3, S4). Using the Stokes–Einstein equation along with a calculated length of one bda‐linker unit of ≈1.59 nm, the DOSY investigations revealed average lengths of 13–14 and 7–8 repeating units for **4 a** and **4 b**, respectively.

**Figure 1:**
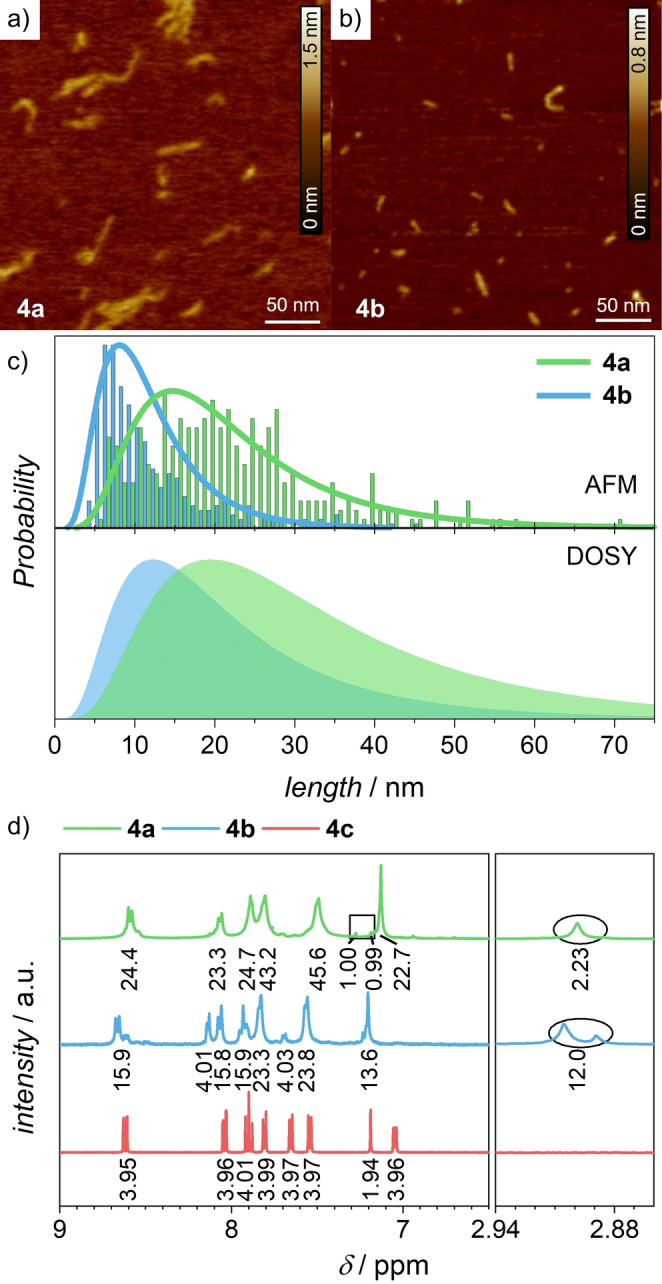
AFM height images after spin‐coating solutions in water/acetonitrile (*ν:ν*, 6 : 4, *c*_Ru_=1.00×10^−5^ M) on mica substrate for a) **4 a** and b) **4 b** (scale bar 50 nm; Z‐scale 1.5 nm (**4 a**) and 0.8 nm (**4 b**)). c) Top: statistical analysis of the oligomer length distribution by visual determination of the length of individual oligomer strands from AFM images. The log‐normal fit of the AFM data is represented as solid lines. Bottom: log‐normal size distribution as obtained from DOSY NMR spectra. d) Excerpt of the ^1^H NMR spectra of the purified materials (400 MHz, CD_3_OD/CD_2_Cl_2_, *ν:ν*, 5 : 1 (**4 a**) or CD_3_OD (**4 b**), 295 K). The end‐groups are marked (square: linker **1**, circle: dmso) and integrals are given below the corresponding signals. Color code: **4 a** (green), **4 b** (blue) and **4 c** (red).

The length distribution was further evaluated visually using atomic force microscopy (AFM) (Figure 1a, b). Samples were prepared by spin‐coating a 6 : 4 (*ν:ν*) water/acetonitrile solution of the oligomers. The AFM images show fiber‐like structures with a height of 0.5±0.5 nm, which is in good accordance with the width of one bda unit perpendicular to the oligomer long axis. Visual determination of the different oligomer lengths are summarized in the histograms shown in Figure 1c (top), which show maxima at 15–22 nm (10–14 repeating units) and 8–9 nm (5–6 repeating units) for **4 a** and **4 b**, respectively. The small deviations in average size obtained by the different techniques can be rationalized in terms of different ways of data acquisition. While for AFM analysis the length of each isolated structure is measured (number average degree of polymerization, see Table S5), the diffusion coefficient is used to determine the weight‐averaged degree of polymerization (see Table S5). In the latter case, larger structures have a higher contribution to the signal, thus resulting in a larger average length of the same sample. Further evidence for the length distribution was obtained by elemental analysis (Figure S4, S5, Table S1, S2) and vapor pressure osmometry (Figure S6–S8). The combined experimental data gave very similar average lengths for **4 a** and **4 b**, thus allowing the determination of the average molar mass for both oligomer mixtures with a high degree of accuracy. These results are summarized in Table S5 and are used for the calculation of the concentration of ruthenium centers throughout the following experiments (see Supporting Information).

The optical and electrochemical properties of **4 a**–**c** were investigated by UV/Vis spectroscopy, cyclic voltammetry (CV) and differential pulse voltammetry (DPV) (Figure S15). In comparison to other oligomers based on the Ru(bda) scaffold,^[35]^ similar absorption features were observed with several ligand‐centered electronic transitions below 330 nm and additional MLCT transitions with a maximum at around 425 nm for the oligomer materials and around 395 nm for **4 c**. In electrochemical investigations, three consecutive oxidation waves were detected at 0.68 V, 0.82 V and 1.03 V for **4 a** and **4 b**. For molecular model compound **4 c**, these potentials are slightly shifted to 0.62 V, 0.84 V and 1.06 V.

Purified samples of WOCs **4 a**–**c** were then employed in visible light‐driven water oxidation (Figure 2). In a three‐component system, varying concentrations of the respective catalyst were combined with Ru(deeb)_2_(bpy)Cl_2_ (bpy=2,2′‐bipyridine, deeb=diethyl 2,2′‐bipyridine‐4,4′dicarboxylate, *c*=0.20 mM)[^19^, ^35^] as photosensitizer and Na_2_S_2_O_8_ (*c=*37.5 mM) as sacrificial electron acceptor (Figure 2a). Irradiation was achieved by a xenon arc lamp equipped with a solar filter at a power of 100 mW cm^−2^. To investigate the effect of both acetonitrile as organic co‐solvent and oligomer length, the initial rate of oxygen evolution was determined for varying concentrations of **4 a**–**c** in solvent mixtures ranging from 20 %–0 % acetonitrile in water (Figure S18).

**Figure 2:**
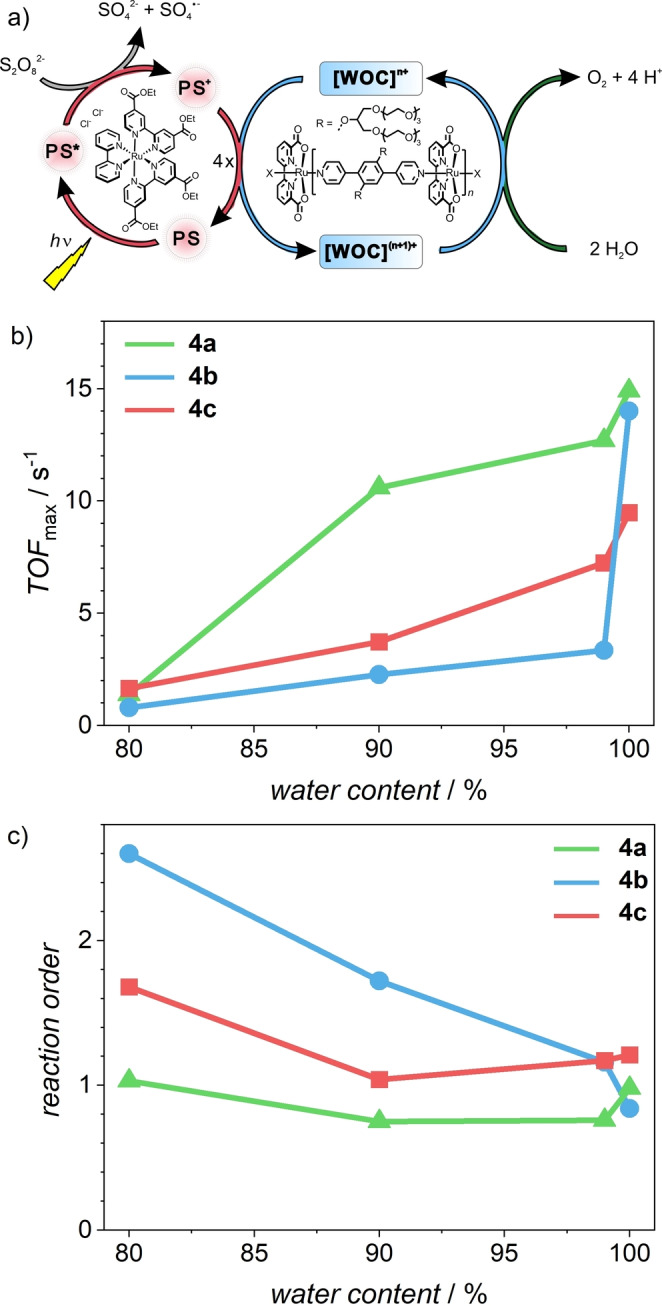
a) Schematic representation of the catalytic cycles for visible light‐driven water oxidation catalysis in a three component system consisting of **4 a**–**c** as water oxidation catalyst, Ru(deeb)_2_(bpy)Cl_2_ as photosensitizer and Na_2_S_2_O_8_ as sacrificial electron acceptor. b) Plot of the TOF_max_ values as a function of the water content during visible light‐driven water oxidation catalysis and c) correlation between the reaction order (determined by linear regression of the plot −log(*d*(*n* ${}_{O{}_{2}}$ )/*dt*) over −log(*c*_Ru_)) and the water content for **4 a** (green), **4 b** (blue) and **4 c** (red).

The maximum turnover frequency TOF_max_ (Table S7) for each catalytic system was plotted as a function of the acetonitrile content to get an overview of the catalyst performance (Figure 2b). To adjust for the different oligomer lengths, all concentrations were normalized to one ruthenium center. As anticipated, all catalysts showed higher activity with decreasing amounts of acetonitrile. Nevertheless, there are decisive differences in the relative activity for the three materials. In pure water, oligomers **4 a** and **4 b** showed the highest activity and stability with TOF_max_ greater than 14 s^−1^ and turnover numbers (TON) of more than 1000. Dinuclear complex **4 c** has a significantly lower performance with a TOF_max_ of 9.5 s^−1^. However, all three materials responded differently to the addition of acetonitrile as a co‐solvent. Reference compound **4 c**, most closely resembling a molecular WOC, shows a gradual decrease in the TOF_max_ upon increasing the acetonitrile fraction in the reaction mixture. This behavior is best described with recent findings about increasing the hydrophobicity of side chains attached to the axial pyridine ligands within molecular WOCs.^[23]^ This hydrophobic effect led to an increase in the observed initial rate of oxygen evolution due to the strengthening of the binding between two individual monomers. Therefore, the diffusion‐controlled formation of a pre‐active dimer, which is the key step for O−O bond formation in the I2M (interaction of two metal oxo radicals, Figure S17) mechanism is preferred for hydrophobic scaffolds.^[37]^ A similar explanation can be drawn for the trend in the initial rate of oxygen evolution for **4 c**. While individual catalysts are well solubilized at higher acetonitrile content, the increasing water fraction destabilizes the solvation shell around the hydrophobic catalysts, hence promoting the formation of dimers and concomitant oxygen evolution.

Similar tendencies might be expected for structurally comparable extended oligomers. For **4 a** however, the TOF_max_ is barely affected by the addition of small amounts of acetonitrile and only drops significantly to 1.4 s^−1^ in the case of 20 % acetonitrile in water. Despite similar connectivity, smaller oligomers **4 b** respond more strongly to the addition of acetonitrile. This can be observed by the sharp drop in activity of **4 b** to 3.4 s^−1^, accompanied by a significant decrease in the TON down to less than 100 after the addition of only 1 % acetonitrile. As the acetonitrile content is further increased, this low activity is continuously reduced. The catalytic performance of oligomeric WOCs **4** in solvent mixtures of varying acetonitrile content largely depends on the oligomer length. To rationalize these findings, a log‐log plot of the initial rate of oxygen evolution against the WOC concentration was constructed to determine the reaction order from the slopes of linear fits for the individual measurements (Figure S20).^[38]^

The plot of the reaction order as a function of the acetonitrile content (Figure 2c) reveals a striking correlation with the TOF_max_ values (Figure 2b). For longer oligomers **4 a**, high TOF_max_ values and a reaction order close to 1 are obtained for the whole range of investigated conditions. By contrast, a continuous transition in the reaction order from around 2 in 20 % acetonitrile to 1 in pure water was observed for the shorter chains in **4 b**. For dimer **4 c**, reaction orders between 1 and 2 are observed for all solvent mixtures. The intricate relation between the reaction order/activity and solvent composition/oligomer length is evident in the plots of the initial rate of oxygen evolution as a function of ruthenium concentration (Figure S19). While **4 a** shows a linear dependency for all investigated conditions, **4 b** undergoes the whole evolution from a quadratic signature when catalysis is carried out with 20 % acetonitrile as co‐solvent to a linear relationship in pure water. For dimer **4 c**, a quadratic contribution is observed for the whole range of solvent mixtures investigated. To quantify these effects, a second‐order polynomial fit was applied to all data to determine the individual contribution of either second or first‐order processes to the overall kinetics of oxygen evolution (Figure 3, Table S6). This unusual observation of a gradual transition in reaction kinetics with varying acetonitrile content naturally raises the question about the exact mechanism of water oxidation for this series of oligomeric WOCs.

**Figure 3:**
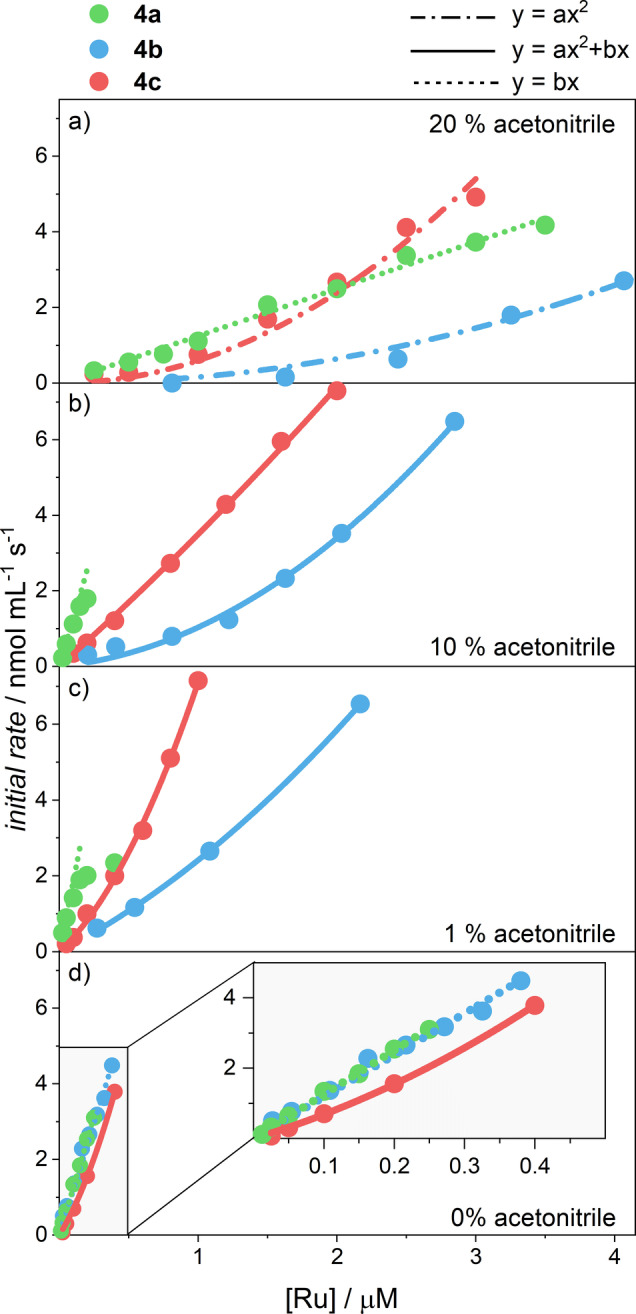
Initial rates of oxygen evolution as determined by linear regression during the first few seconds of water oxidation catalysis in aqueous phosphate buffer (colored dots, **4 a** (green), **4 b** (blue), **4 c** (red)) containing a) 20 %, b) 10 %, c) 1 % and d) 0 % acetonitrile as co‐solvent. The experimental data points were fitted to either a second order polynomial (solid line), quadratic (dash‐dotted line) or linear (dotted line) equation.

To determine if there is a fundamental change in the reaction mechanism, H/D kinetic isotope effect (KIE) experiments were carried out. For the I2M pathway as the major mechanism for Ru(bda)‐type catalysts, values around 1 are expected (i.e., secondary KIE) since no proton transfer is involved in the rate‐limiting step.^[37]^ If the catalysts would follow a single‐site water nucleophilic attack (WNA) pathway, however, values around 2 (i.e., primary KIE) would be observed due to the deprotonation associated with the nucleophilic attack of the water molecule (For details see Figure S17). Performing these experiments under neutral conditions, a secondary KIE with experimental values in the range of 0.8–1.3 was observed for **4 a**–**c** irrespective of the acetonitrile content (Figure S21). Based on these results, it can be excluded that the change in reaction kinetics is induced by a change in mechanism from I2M to WNA at higher water content.

Considering all data, we propose the following explanation for the observed transition from second to first‐order kinetics while maintaining the I2M mechanism. On the one hand, higher acetonitrile content and short oligomers favor complete dissolution into single oligomer chains, which requires a diffusion‐controlled dimer formation during oxygen formation. On the other hand, with increasing chain length and decreasing acetonitrile content, there is a high tendency for the linear oligomers to self‐assemble into larger superstructures. Due to the very high local concentration of ruthenium centers in the self‐assembled oligomer fibers, the diffusion‐controlled bimolecular process is replaced by a much faster *intra*‐assembly oxygen evolution reaction, as indicated by first‐order kinetics for this process (Figure 4).

**Figure 4:**
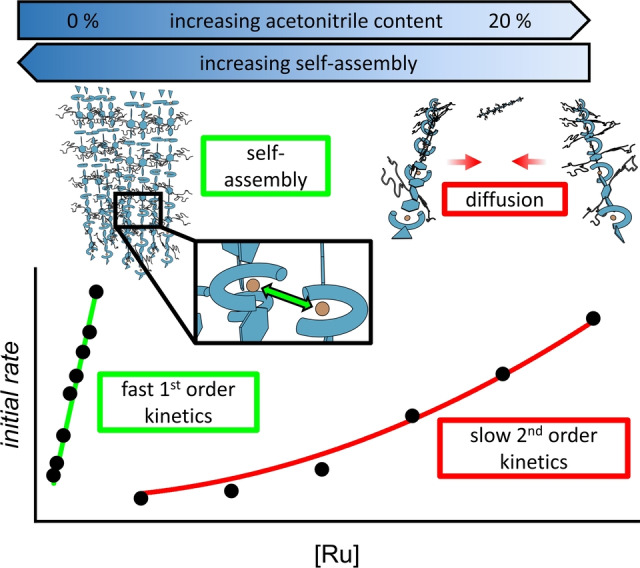
Schematic illustration of the different pathways for O−O bond formation for medium‐sized oligomers **4 b** by *intra*‐assembly I2M mechanism with first order kinetics in pure water (left) or diffusion‐controlled dimer formation between oligomer strands with second order kinetics in 20 % acetonitrile (right).

For **4 a**, first‐order kinetics are already observed for measurements in 20 % acetonitrile in water (Figure 3a, green plot). This is in line with the AFM images obtained after spin‐coating a solution of **4 a** in 6 : 4 (*ν:ν*) water/acetonitrile on mica substrate (Figure 1a). Even for this large amount of organic co‐solvent, many self‐assembled oligomer structures are observed, thus indicating a strong tendency for **4 a** to self‐assemble into extended structures. Upon further decreasing the acetonitrile content in the catalytic mixture, distinct first‐order kinetics are maintained in combination with a highly augmented catalytic activity as compared to **4 b**. In contrast, AFM images of **4 b** spin‐coated from a 6 : 4 (*ν:ν*) water/acetonitrile mixture on mica substrate showed only isolated oligomer fibers (Figure 1b). These findings corroborate the increasing tendency for self‐assembly upon elongation of the oligomers. Naturally, a stronger driving force for self‐assembly is also expected for decreasing acetonitrile content in the solvent mixture. Indeed, this was observed for both **4 a** and **4 b** in AFM images spin‐coated from an aqueous solution on mica (Figure S12, S13). While almost exclusively insular structures without concurrent isolated fibers in the images for **4 a** indicate very efficient self‐assembly, significantly smaller superstructures are observed for **4 b**. However, the pronounced aggregation of **4 a** comes with the drawback of lower solubility in pure water for this material. Instead, the smaller oligomers **4 b** are significantly more soluble while still maintaining the very high activity of WOC aggregates without the addition of organic solvents. Therefore, it can be inferred that the implementation of molecular catalysts into metallosupramolecular oligomers significantly increases both the activity and the stability of visible light‐driven WOCs in aqueous environment.

For longer oligomers, self‐assembly into larger superstructures results in efficient catalytic systems even in mixtures containing up to 20 % acetonitrile as co‐solvent. For shorter oligomers, solvent mixtures with less acetonitrile content are required for the efficient formation of highly active assemblies. In the case of pure water, both **4 a** and **4 b** are self‐assembled into supramolecular WOCs with excellent performance and stability in visible light‐driven water oxidation catalysis compared to previously published systems (Table S9, Figure S22). Overall, medium‐sized oligo–mers **4 b** proved to be excellent materials for water oxidation as they combine high‐yielding synthesis and easy purification with high water solubility and excellent catalytic performance as well as stability due to pronounced self‐assembly in aqueous solution.

In summary, a series of multinuclear Ru(bda) complexes **4 a**–**c** with varying chain length was synthesized by adjusting the ratio between the bipyridine linker **1** and ruthenium precursor **2** or **3**, respectively. For extended oligomers bearing more than five ruthenium centers, visible light‐driven water oxidation catalysis in pure water afforded unprecedented oxygen evolution even at nanomolar concentrations with excellent efficiencies of up to 14.9 s^−1^ and more than 1000 turnovers per Ru center. This high catalytic efficiency was attributed to the formation of a self‐assembled catalytic nanosystem with a very high effective concentration of individual ruthenium centers. Thereby, the formation of active dimers in the I2M mechanism is not diffusion‐controlled but rather facilitated via highly efficient supramolecular preorganization.

## Conflict of interest

The authors declare no conflict of interest.

## Supporting information

As a service to our authors and readers, this journal provides supporting information supplied by the authors. Such materials are peer reviewed and may be re‐organized for online delivery, but are not copy‐edited or typeset. Technical support issues arising from supporting information (other than missing files) should be addressed to the authors.

Supporting Information

## Data Availability

The data that support the findings of this study are available in the Supporting Information of this article.
