# Supplementary material for: Self‐assembled Ru(bda) Coordination Oligomers as Efficient Catalysts for Visible Light‐Driven Water Oxidation in Pure Water
Source: Angew Chem Int Ed Engl. 2022 Nov 23;61(52):e202211445. doi: 10.1002/anie.202211445 (PMC10100213; doi:10.1002/anie.202211445)
Supplement: Supplementary file 1 — Supporting Information [file ANIE-61-0-s001.pdf]

Angewandte Chemie
Eine Zeitschrift der Gesellschaft Deutscher Chemiker

Supporting Information

Self-assembled Ru(bda) Coordination Oligomers as Efficient Catalysts for Visible Light-Driven Water Oxidation in Pure Water

*T. Schlossarek, V. Stepanenko, F. Beuerle, F. Würthner**

Supporting Information

Table of contents

Materials and methods	2
Synthesis	5
Oligomer characterization	9
Vapor pressure osmometry	15
Optical and electrochemical properties	24
Visible light-driven water oxidation catalysis	25
Analytical data for molecular precursors and Ru(bda) oligomers	34
Literature	39

Materials and methods

General

All reagents and solvents were purchased from commercial sources and used without further purification. 2,5-Dibromo-1,4-hydroquinone (**S1**),[S1] tosylated oligoethyleneglycol precursor **S2**,[S2] 4-pyridylboronic acid pinacol ester (**S4**),[S3] Ru(bda)(dmso)$_2$ (**2**)[S4] and Ru(bda)(dmso)(4-pic) (**3**)[S5] were synthesized according to published procedures. Anhydrous methanol was obtained by refluxing over CaH_2 and subsequent distillation on molecular sieves 3 Å.

NMR spectroscopy

^{1}H NMR and ^{13}C NMR spectra were recorded on an Avance III HD 400 spectrometer (*Bruker Daltonics GmbH*) operating at a temperature of 295 K and a frequency of 400 MHz (^{1}H) and 100 MHz (^{13}C), respectively. Chemical shifts (δ) are stated in parts per million (ppm) using the residual solvent signal for calibration against TMS and coupling constants J are given in Hz. Multiplicities are described as singlet (s), doublet d), triplet (t) or multiplet (m).

Mass spectrometry

High-resolution ESI-TOF measurements were obtained on an ESI MicrOTOF focus mass spectrometer (*Bruker Daltonics GmbH*).

Elemental analysis

Elemental analysis was performed on a Vario MICRO cube (*Elementar Analysensysteme GmbH*).

Flash column chromatography

If not stated otherwise, compound purification was achieved using an automated flash purification system PuriFlash 420 (*Interchim*) using pre-packed silica gel columns (*Interchim*) with a grain size of 30 µm and distilled solvents.

Electrochemistry

Cyclic voltammetry (CV) and differential pulse voltammetry (DPV) measurements were performed on a BAS Cell Stand C3 (*BAS Epsilon*) using a three-electrode setup with glassy carbon as working electrode, platinum wire as counter electrode and Ag/AgCl (3 M KCl) as reference electrode. Voltammograms were recorded at a scan speed of 100 mV s^{-1}. The experiments were carried out in neutral aqueous phosphate buffer (0.1 M ionic strength at

pH = 7.0) as solvent. The obtained potentials were converted to normal hydrogen electrode by addition of +0.21 V.[S6]

UV/vis absorption spectroscopy

UV/vis spectra were recorded on a V-770 UV/vis/NIR spectrophotometer (*Jasco Inc*) at 298 K using Suprasil quartz cells with a path-length of 1 cm. Solutions were prepared using spectroscopy grade solvents (acetonitrile) or doubly distilled water that was obtained by passing distilled water through a Millipore filter system. Absorptivities are converted into extinction coefficients using the Beer-Lambert law.

Visible light-driven water oxidation catalysis

Visible light-driven water oxidation experiments were performed using an Oxygraph Plus Clark-electrode system (*Hansatech Instruments Ltd.*) for oxygen detection. Sample irradiation was achieved by a 150 W xenon arc lamp (*Newport*) equipped with a 400 nm cutoff filter. The light intensity was calibrated to 100 mW cm^{-2} using a PM 200 optical power meter with a S121C sensor (*Thorlabs Inc*) in combination with a CCS 200/M wide range spectrometer (*Thorlabs Inc*). Experiments were conducted using a stock solution of photosensitizer Ru(deeb)$_2$(bpy)Cl$_2$ (c = 0.27 mM) and sacrificial electron acceptor $Na_2S_2O_8$ (c = 50 mM) in mixtures of aqueous phosphate buffer (pH = 7.2, *Honeywell Fluka*) and acetonitrile in different ratios. An aliquot of the respective stock solution (V = 1.5 mL) was transferred to a transparent, water-cooled reaction chamber (T = 293 K) and mixed with a catalyst solution in the same solvent mixture while kept in the dark. The sample volume was adjusted with pure solvent mixture to reach 2 mL. Irradiation was initiated after 40 seconds to allow thermal equilibration of the sample at 20 °C. The TOF was obtained by linear regression of the initial rate of oxygen evolution during the first few seconds of catalysis. The TON was calculated based on the maximum amount of oxygen evolved during catalysis divided by the amount of used catalyst. The highest TON obtained from concentration-dependent experiments is reported.

Kinetic isotope effect experiments

H/D Kinetic isotope effect experiments were carried out under similar conditions as described for the visible light-driven water oxidation catalysis. Aqueous phosphate buffer solutions (50 mM) were prepared by dissolving 340 mg of KH_2PO_4 in 50 mL of H_2O or D_2O, respectively. The pH(D) was adjusted by addition of ~2.0 mL of a solution of 1 M NaOH and 50 mM KH_2PO_4 in H_2O or D_2O. The pH was monitored using a pH electrode (*Mettler Toledo*) after two point calibration.

Vapor pressure osmometry

VPO was measured using a K-7000 vapor pressure osmometer (*Knauer GmbH*) equipped with a universal temperature measurement unit using chloroform as solvent at a temperature of 313 K. Benzil and two linear polystyrene standards of narrow molecular weight distributions (*Polymer Standards Service GmbH*) were used to generate calibration curves. Each experiment was conducted at least twice to obtain reproducible values.

DOSY

DOSY measurement were carried out in a mixture of CD_2Cl_2 and CD_3OD (*v:v*, 1:5) on an Avance III HD 600 or an Avance III HD 400 spectrometer (*Bruker Daltonics GmbH*) operating at a frequency of 600 MHz or 400 MHz, respectively. Experiments were conducted using DSTE and LED pulse sequence with a diffusion time of 100 ms. The decay curves were evaluated according to the log-normal model[S7–S8] as described below.

Atomic force microscopy

AFM measurements were performed under ambient conditions using a Multimode 8 SPM system (*Bruker Daltonics GmbH*) operating in tapping mode under air. Silicon cantilevers (*OMCL-AC240TS, Olympus*) with a resonance frequency of ~70 kHz and a spring constant of ~1.7 Nm^{-1} were used. For preparation of AFM samples, the solution of **4a** and **4b** in water or water/acetonitrile (*v:v*, 6:4) with a concentration of ruthenium centers of 1.00×10^{-5} M was spin-coated onto mica with 7000 rpm. Statistical analysis of the length distribution was performed using the program NanoScope Analysis 1.5 (*Bruker*).

Molecular modelling

Molecular modelling was performed using the program Materials Studio (*Biovia*).[S9] Structural models were energy-minimized with the Universal Force-field (UFF) with a cut-off distance of 12.5 Å by using the Forcite Task.

Synthesis

Ru(bda) oligomers **4a** and **4b** were obtained in a multi-step procedure starting with the functionalization of 2,5-dibromo-1,4-hydroquinone (**S1**) with the water-solubilizing sidechains. Subsequent Suzuki coupling gave linker precursor **1** in 75 % yield. Finally, polymerization of ditopic ligand **1** and Ru(bda)(dmso)$_2$ (**2**) in anhydrous and deaerated methanol was carried out in different ratios to give the desired oligomers in more than 70 % yield for **4a** and above 90 % yield for **4b**, respectively. For **4c**, linker **1** was reacted with an excess of Ru(bda)(dmso)(4-pic) (**3**) to give the desired compound in 10 % yield.

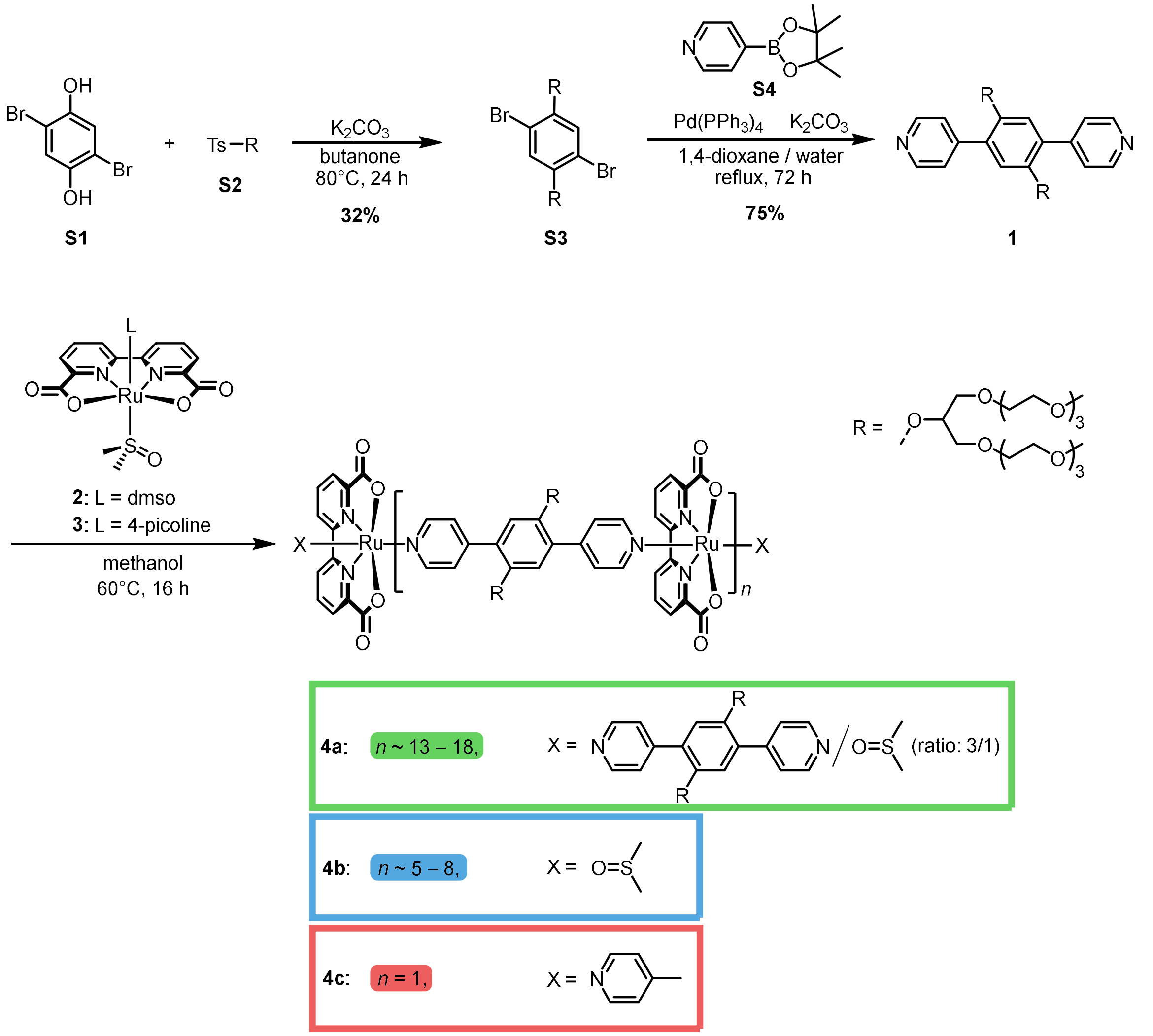

Scheme S1. Synthetic scheme for the synthesis of **4a–c**.

Experimental procedures

13,13'-((2,5-Dibromo-1,4-phenylene)bis(oxy))bis(2,5,8,11,15,18,21,24-octaoxapentacosane) (**S3**)

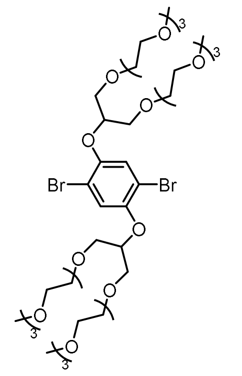

Chemical Formula: $C_{40}H_{72}Br_2O_{18}$
Molecular Weight: 1000.8060

2,5-Dibromo-1,4-hydroquinone (**S1**) (500 mg, 1.87 mmol, 1.0 eq) and tosylate **S2** (2.21 g, 4.11 mmol, 2.2 eq) are dissolved in butanone (10 mL) and the solution is degassed. Afterwards, K_2CO_3 (1.55 g, 11.2 mmol, 6.0 eq) is added and the mixture is stirred at 80 °C for 24 h. After cooling to room temperature, the mixture is filtrated over celite (dichloromethane as eluent) and purified by column chromatography on silica (gradient elution using dichloromethane and 0 %–2 % methanol as eluent).

Yield: 599 mg (599 µmol, 31 %) of a light-yellow oil.

^{1}H NMR (400 MHz, $CDCl_3$, 295 K): δ = 7.35 (s, 2H, Ar*H*), 4.41–4.35 (m, 2H, OC*H*), 3.74–3.62 (m, 48H, OCH_2), 3.55–3.53 (m, 8H, OCH_2) 3.37 (s, 6H, OCH_3) ppm.

^{13}C NMR (100 MHz, $CDCl_3$, 295 K): δ = 150.7, 122.1, 112.8, 80.9, 72.1, 71.3, 70.9, 70.8, 70.7, 59.2 ppm.

HRMS (ESI-TOF, positive mode, MeCN): *m/z* calculated for $C_{40}H_{72}Br_2NaO_{18}$ $[M+Na]^+$: 1023.2958; found 1023.2914.

4,4'-(2,5-Bis((2,5,8,11,15,18,21,24-octaoxapentacosan-13-yl)oxy)-1,4-phenylene)dipyridine (**1**)

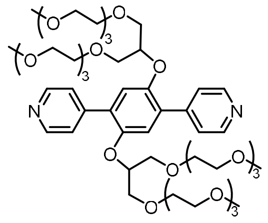

Chemical Formula: $C_{50}H_{80}N_2O_{18}$
Molecular Weight: 997.1860

Dibromide **S3** (250 mg, 250 µmol, 1.0 eq), 4-pyridylboronic acid pinacol ester (**S4**) (128 mg, 635 µmol, 2.5 eq) and K_2CO_3 (138 mg, 999 µmol, 4.0 eq) are dissolved in a degassed mixture of 1,4-dioxane (10 mL) and water (2 mL). Afterwards, $Pd(PPh_3)_4$ (28.9 mg, 25.0 µmol, 0.1 eq) is added and the mixture is heated under reflux for 72 hours. After cooling to room temperature, water (20 mL) is added, and the mixture is extracted with dichloromethane (6 × 70 mL). The combined organic layers are dried over sodium sulfate and evaporated. The crude compound is purified by column chromatography on silica (gradient elution using dichloromethane and 0 %–10 % methanol as eluent).

Yield: 188 mg (189 µmol, 74 %) of a yellow oil.

^{1}H NMR (400 MHz, $CDCl_3$, 295 K): δ = 8.65–8.64 (m, 4H, Ar$H_{pyridine}$), 7.64–7.63 (m, 4H, Ar$H_{pyridine}$), 7.22 (s, 2H, Ar*H*), 4.49–4.44 (m, 2H, C*H*), 3.61–3.50 (m, 48H, OC*H*2), 3.35 (s, 12H, OCH_3) ppm.

^{13}C NMR (100 MHz, $CDCl_3$, 295 K): δ = 150.5, 149.2, 146.3, 130.3, 124.6, 118.3, 79.4, 72.0. 71.0, 70.7, 70.6, 59.1 ppm.

HRMS (ESI-TOF, positive mode, MeCN): *m/z* calculated for $C_{50}H_{81}NaN_2O_{18}$ $[M+Na]^+$: 1019.5299; found 1019.5259.

Ru(bda) oligomer **4a**

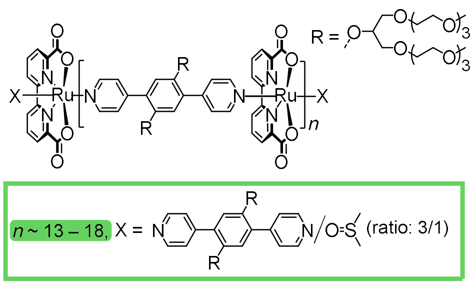

Bipyridine linker **1** (100 mg, 100 µmol, 1.15 eq) and Ru(bda)(dmso)$_2$ (**2**) (43.6 mg, 87.2 µmol, 1.00 eq) are dissolved in anhydrous methanol (10 mL) and the mixture is deaerated with nitrogen. Afterwards, the mixture is heated at 60 °C over a period of 16 h. After cooling to room temperature, the solvent is evaporated and the crude compound is purified by three repetitive precipitations from chloroform with *n*-hexane.

Yield: 88.0 mg (64.1 µmol, 74 %) of a red-brown solid.

^{1}H NMR (400 MHz, CD_3OD/CD_2Cl_2 (5:1), 295 K): δ = 8.62–8.51 (br, 24H, Ar*H*), 8.15–8.02 (m, 24H, Ar*H*), 7.92–7.74 (br, 68H, Ar*H*), 7.71–7.35 (br, 48H, Ar*H*), 7.26 (s, 1H, Ar*H*), 7.17 (s, 1H, Ar*H*), 7.16–7.06 (m, 22H, Ar*H*) , 4.47–4.39 (br, 24H, C*H*), 3.54–3.39 (m, 672H, OCH_2), 3.29–3.26 (br, 137H, OCH_3), 2.90 (s, 2H, DMSO-CH_3) ppm.

Elemental analysis calculated (%) for $C_{2160}H_{3010}N_{138}O_{767}Ru_{34}S$: C 55.61, H 6.50, N 4.14, S 0.07; found, C 55.14, H 6.61, N 4.02.

Ru(bda) oligomer **4b**

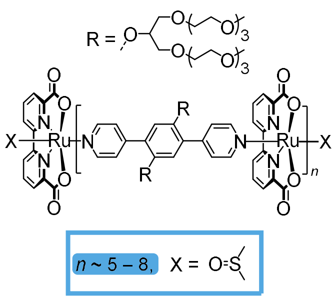

Bipyridine linker **1** (50.0 mg, 50.1 µmol, 1.0 eq) and Ru(bda)(dmso)$_2$ (**2**) (25.2 mg, 50.1 µmol, 1.0 eq) are dissolved in anhydrous methanol (5 mL) and the mixture is deaerated with nitrogen. Afterwards, the mixture is heated at 60 °C over a period of 16 h. After cooling to room temperature, the solvent is evaporated and the crude compound is purified by three repetitive precipitations from chloroform with *n*-hexane.

Yield: 66.0 mg (48.1 µmol, 96 %) of a red-brown solid.

^{1}H NMR (400 MHz, CD_3OD, 295 K): δ = 8.78–8.59 (br, 16H, Ar*H*), 8.16–7.80 (m, 20H, Ar*H*), 7.98–7.77 (br, 20H, Ar*H*), 7.71–7.66 (m, 4H, Ar*H*), 7.64–7.48 (m, 24H, Ar*H*), 7.27–7.14 (m, 14H, Ar*H*), , 4.50–4.39 (br, 14H, C*H*), 3.54–3.35 (m, 392H, OCH_2), 3.22 (br, 84H, OCH_3), 2.90 (s, 12H, dmso-CH_3) ppm.

Elemental analysis calculated (%) for $C_{450}H_{620}N_{30}O_{160}Ru_8S_2$: C 54.69, H 6.32, N 4.25, S 0.65; found, C 54.28, H 6.67, N 4.28, S 0.52.

Ru(bda) dimer **4c**

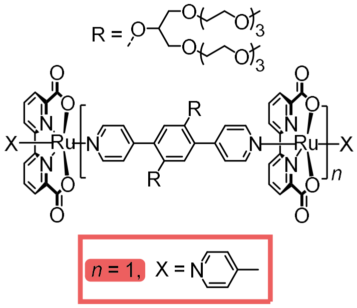

Bipyridine linker **1** (40.0 mg, 40.1 µmol, 1.0 eq) and Ru(bda)(dmso)(4-pic) (**3**) (47.7 mg, 92.3 µmol, 2.3 eq) are dissolved in anhydrous methanol (5 mL) and the mixture is deaerated with nitrogen. Afterwards, the mixture is heated at 60 °C over a period of 16 h. After cooling to room temperature, the solvent is evaporated and the crude compound is purified by column chromatography on silica (DCM with 10 %–20 % MeOH as eluent) and subsequent column chromatography on Alox (Act. V, DCM with 0 %–2.5 % MeOH as eluent).

Yield: 7.70 mg (4.11 µmol, 10 %) of a red-brown solid.

^{1}H NMR (400 MHz, CD_3OD, 295 K): δ = 8.62 (dd, ^3J = 8.1 Hz, ^4J = 1.0 Hz, 4H, Ar*H*), 8.05 (dd, ^3J = 7.8 Hz, ^4J = 0.9 Hz, 4H, Ar*H*), 7.91 (t, ^3J = 7.9 Hz, 4H, Ar*H*), 7.83–7.81 (m, 4H, Ar*H*), 7.67–7.66 (m, 4H, Ar*H*), 7.56–7.55 (m, 4H, Ar*H*), 7.20 (s, 2H, Ar*H*), 7.07–7.05 (m, 4H, Ar*H*), 4.47–4.42 (m, 2H, C*H*), 3.55–3.34 (m, 56H, OCH_2), 3.23 (s, 12H, OCH_3), 2.27 (s, 6H, CH_3) ppm.

^{13}C NMR (100 MHz, CD_3OD, 295 K): δ = 175.1, 161.6, 157.6, 152.6, 152.3, 151.8, 150.9, 147.7, 133.0, 130.1, 127.1, 126.8, 126.3, 118.9, 80.3, 72.9, 71.8, 71.6, 71.5, 71.3, 59.1, 20.7 ppm.

HRMS (ESI-TOF, positive mode, MeCN): *m/z* calculated for $C_{86}H_{106}N_8O_{26}Ru_2$ $[M+Na]^+$: 1870.5300; found 1870.5438.

Oligomer characterization

Oligomer formation process

To assign proton signals in the ^{1}H NMR spectra of the final oligomers, the formation process was monitored by heating an equimolar ratio of Ru(bda)(dmso)$_2$ and ditopic pyridine linker **1** in CD_3OD at 60 °C and measuring time-dependent NMR spectra. Generally, three different cases of linker positioning within the oligomer need to be considered and can be distinguished by different chemical shifts in the spectrum:

1) The linker is only coordinated to a ruthenium center with one nitrogen atom (blue case, end-group)
2) The linker is coordinated to a ruthenium center opposite of a dmso ligand (red case)
3) The linker is coordinated on both sides to the ruthenium center with opposing pyridine ligands (green case, central unit)

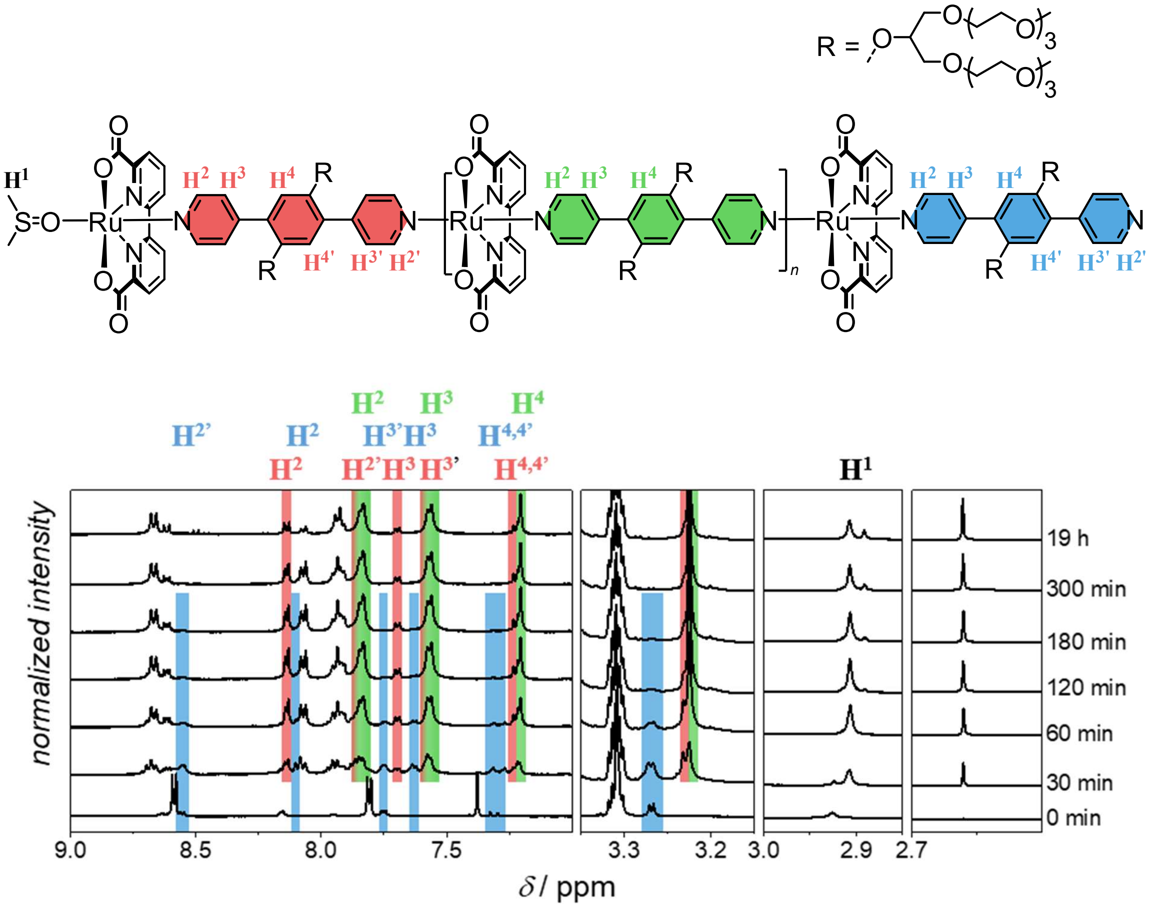

Figure S1. Chemical structure of an oligomer based on linker **1** and Ru(bda)(dmso)$_2$ and the three different cases of linker positions within the oligomer. Bottom: Time-dependent ^{1}H NMR

spectra (400 MHz, CD_3OD, 295 K) to monitor the oligomer formation process and assign signals according to the placement of the linker within the oligomer.

Detailed analysis of the NMR spectrum gives a clear indication for assignment of the signals to the three cases described above. In the initial spectrum (blue curve), signals of the linker unit are almost exclusively visible due to bad solubility of the Ru(bda)(dmso)$_2$ complex in methanol at room temperature. Upon heating, the formation of the oligomer can be observed and NMR signals assigned to the three different ligand placements arise. For reasons of simplification, only signals belonging to linker **1** are discussed in this summary:

1) Distinctive for the blue end-group ligand is the fact that only one nitrogen atom is coordinated to the ruthenium center leading to one pronounced high-field shift of aromatic pyridine protons of **1** while the signal of the protons attached to the non-coordinated pyridine do not show a significant shift. These pyridine protons additionally split into four different signals due to the different environment upon one-fold coordination to ruthenium. Further splitting of the signal belonging to the protons at the central benzene moiety supports the fact of the linker placement on the outer part of the oligomer. In the beginning of the reaction, **no** prediction can be made whether these signals belong to a linker species coordinated to a ruthenium center with a dmso or another linker **1** ligand on the other coordination site, making this assignment only conclusive after the reaction is carried out.

   Other protons exhibiting NMR signals indicative for this case belong to the methyl groups of the OEG chains. This signal is slightly shifted to higher fields compared to the free linker and also splits into two separate singlets.
2) After short reaction time, signals belonging to **1** coordinated on one side opposite to dmso and on the other side opposite of another **1** molecule (red case) become visible. Here, a more pronounced high-field shift compared to the blue case can be observed. However, only signals for the protons attached to the pyridine ligand opposing the dmso ligand are visible while signals belonging to the other pyridine ligand get mixed within the signals belonging to the core units of the oligomer (green case) due to their electronic similarity. The existence of dmso end-groups can be extracted from the signal at 2.90 ppm belonging to the bound species. Free dmso arising during the reaction progress, can in contrast be observed at chemical shifts of around 2.65 ppm.

3) Furthermore, signals belonging to the central units increase after short reaction time and feature broad signals without a defined structure. All signals are shifted to the high-field region compared to the other cases.

Subsequently, additional free linker **1** in CD_3OD solution was added to the NMR tube and the reaction was carried out for 5 h at 60 °C before another NMR spectrum was recorded. This method proved useful to assign signals to the respective cases where the linker units are located on the outer part of the oligomer strand (red and blue case in Figure S1).

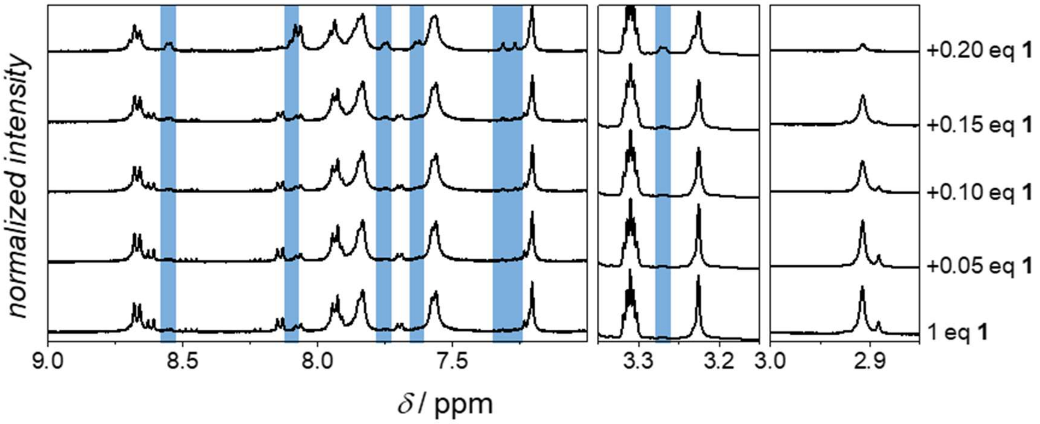

Figure S2. ^{1}H NMR spectra (400 MHz, CD_3OD, 295 K) of the final oligomer described in Figure S1 (blue curve) and spectra measured after addition of linker **1** and subsequent reaction for 5 h at 60 °C in CD_3OD. The blue rectangles mark signals belonging to the blue case as described in Figure S1.

In the spectrum, the signals belonging to **1** as end-group are marked in blue. Upon addition of 0.20 eq of linker **1**, the rise of six signals in the aromatic region become visible. This is a result of the one-fold coordination to a ruthenium center and concomitant splitting of the NMR signals. Additionally, two signals rise for the methyl groups of the OEG chain at around 3.28 ppm. Simultaneously, the signal assigned to dmso as end-group keeps decreasing while linker is added to the mixture indicating the efficient transformation of the end-groups when free pyridine ligands are added to the solution.

End-group analysis

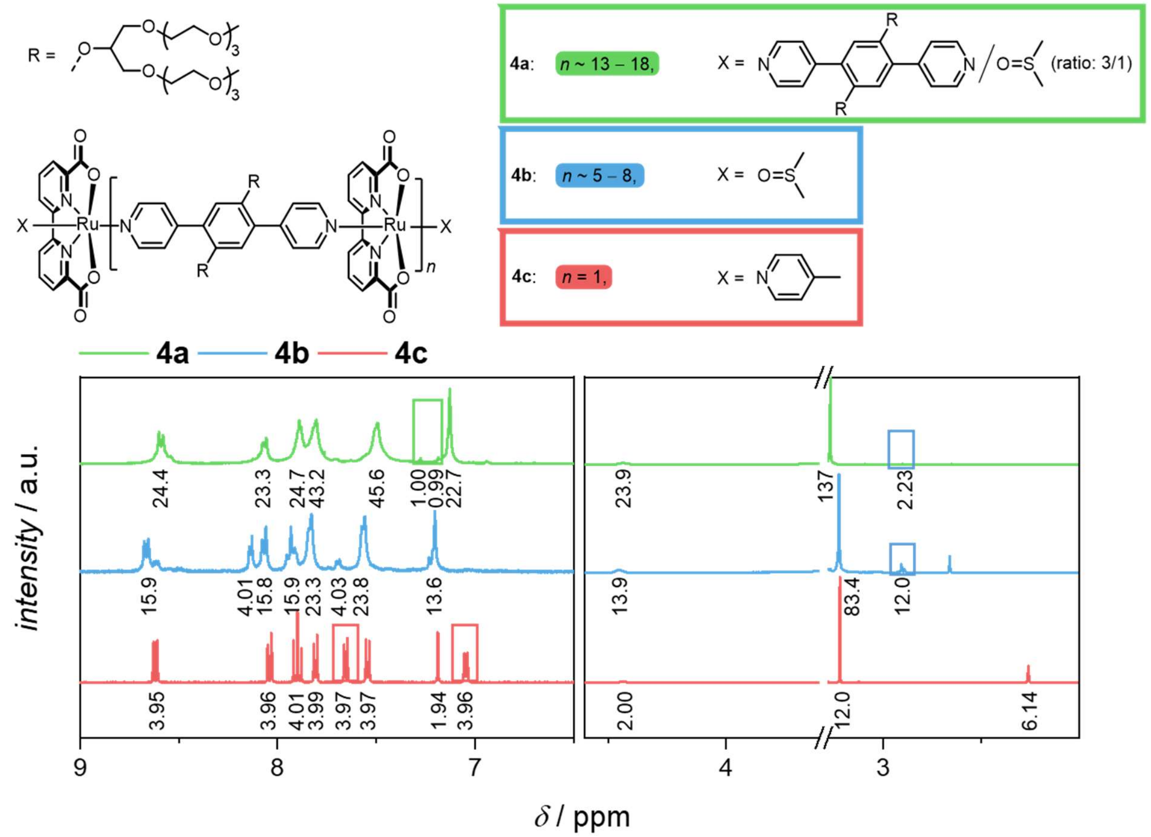

Figure S3. Molecular structures of **4a–c**. ^{1}H NMR spectrum of the purified materials (400 MHz, CD_3OD or CD_3OD/CD_2Cl_2, *v:v*, 5:1, 295 K). Green line: **4a**, blue line: **4b**, red line: **4c**. The end-groups are marked with the respective colored squares and numbers below spectra represent the corresponding integrals of the signals.

End-group analysis via integration of specific NMR signals of the terminal groups (Figure S3, rectangles, red: 4-picoline, green: linker **1**, blue: dmso) in reference to specific NMR signals of the inner bipyridine linkers and bda units allow an estimation of the average amount of repeating units per oligomer chain. For **4c**, the dinuclear structure was confirmed by this analysis. For oligomeric mixtures **4a** and **4b**, this approach was used in the calculation of the concentration of ruthenium centers during measurements and catalysis.

For **4b**, the integral of the dmso end-groups at δ = 2.90 ppm was set to 12 corresponding to the amount of protons for two individual ligands. The integration of signals belonging to linker unit **1** (δ = 4.45 ppm, C*H* protons of the OEG chain, δ = 7.20 ppm, aromatic protons of the central benzene moiety) results in the estimation of 7 linker units per oligomer strand. The integration of NMR signals indicative for the bda moiety (e.g. δ = 7.72 ppm) gave slightly larger values of around 16 revealing the occurrence of 8 bda units in the strand. The molecular weight of an

oligomer composed of these moieties results in a value of 9882 g mol^{-1}. The average molecular weight per ruthenium center is afterward obtained by dividing by the amount of bda units in the strand resulting in an average molecular weight per ruthenium center of around M_{Ru} ~ 1235 g mol^{-1} for **4b** (Figure S3, blue spectrum).

This value is different in the case of **4a** due to the occurrence of different end-groups in the oligomer strand (*vide supra*). The presence of end-groups consisting of **1** and dmso units is suggested by observation of NMR signals at 7.26 ppm and 7.17 ppm (I = 1.00, splitting of the protons of the central benzene unit due to the different chemical environment on the outside of the oligomer strand, see Figure S2). Additionally, a NMR signal at 2.90 ppm indicates the presence of dmso as end-group. According to integration of the signals, the ratio between the end-groups is 3:1 (linker **1**:dmso). The C*H* protons of the OEG chain and the protons of the central benzene moiety of the linker unit both integrate to 24 protons. Considering the different end-groups, this results in an average amount of 17–18 linker units in the oligomer (depending on the end-group). Signals representative for the bda unit feature integrals of 23–24 protons in Figure S3. This results in an average amount of 17–18 bda units in the oligomer strand and an average molecular weight of 23325 g mol^{-1} per oligomer strand. Dividing by the amount of ruthenium centers per oligomer strand (i.e. 17), results in an average molecular weight per ruthenium center of M_{Ru} = 1372 g mol^{-1}.

Elemental analysis

4a

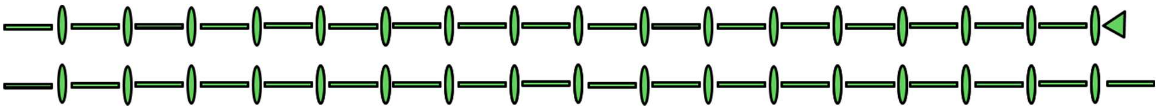

Figure S4. Schematic representation of two oligomers consisting of 17 Ru(bda) and 16 linker units and dmso and linker end-groups in a 1:3 ratio as estimated by ^{1}H NMR spectroscopy of **4a** (rectangle: linker **1**, oval: Ru(bda), triangle: dmso).

Table S1. Calculated elemental composition for **4a** consisting of 17 Ru(bda) and 16 linker units and dmso and linker end-groups in a 1:3 ratio and experimental values from elemental analysis. The length and nature of end-groups of **4a** were estimated by ^{1}H NMR spectroscopy.

	C	H	N	O	Ru	S
Calculated $C_{2160}H_{3010}N_{138}O_{767}Ru_{34}S$	55.61	6.50	4.14	26.30	7.37	0.07
Found	55.14	6.61	4.02	-	-	- [a]

[a] No signal observed due to the sensitivity of the device used.

4b

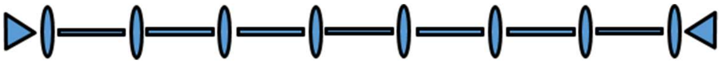

Figure S5. Schematic representation of an oligomer consisting of two dmso end-groups, eight Ru(bda) and seven linker units as estimated by ^{1}H NMR spectroscopy of **4b** (rectangle: linker **1**, oval: Ru(bda), triangle: dmso).

Table S2. Calculated elemental composition for **4b** consisting of two dmso end-groups, eight Ru(bda) units and seven linker units and experimental values from elemental analysis. The length and nature of end-groups of **4b** were estimated by ^{1}H NMR spectroscopy.

	C	H	N	O	Ru	S
Calculated $C_{450}H_{620}N_{30}O_{160}Ru_8S_2$	54.69	6.32	4.25	25.90	8.18	0.65
Found	54.28	6.67	4.28	-	-	0.52

Vapor pressure osmometry

Further investigation of the average number of repeating units was carried out by vapor pressure osmometry. For determination of the calibration constant, benzil and two linear polystyrene GPC standards were measured at 313 K in chloroform.

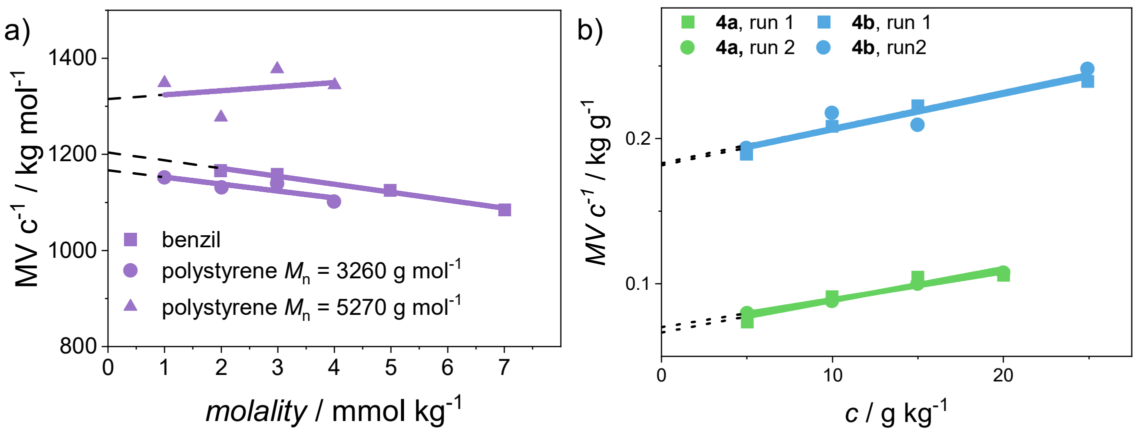

Figure S6. a) *MV/m* against molality plot obtained during VPO measurements of benzil (purple squares) and polystyrene samples (M_n =3260 g mol^{-1}, purple dots; M_n = 5270 g mol^{-1}, purple triangles). The solid purple lines correspond to the linear fit of the obtained data points and the black dotted lines show the respective extrapolation to m = 0 mmol kg^{-1}. b) *MV/c* against concentration of **4a** (green symbols) and **4b** (blue symbols) obtained during VPO measurements of Ru(bda) oligomers. Two individual measurements are shown for both oligomers. The solid lines show the corresponding linear fits of the obtained data points and the black dotted line the extrapolation to c = 0 g kg^{-1}. The average value was used for calculation of the number average molecular weight.

The extrapolation of the linear fit of the measurement data results in the following values for the calibration constants K_{cal}:

Benzil: K_{cal} = 1204.26 kg mol^{-1}

PS3260: K_{cal} = 1166.99 kg mol^{-1}

PS5270: K_{cal} = 1315.49 kg mol^{-1} .

The average of these values is K_{cal} = 1228.91 kg mol^{-1} which is used in the determination of the number average molecular weight of the oligomers. Two individual measurements of **4a** solutions of different concentrations and extrapolation of the linear fit to c = 0 g kg^{-1} gives an average value for K_{meas} = 0.0682 kg g^{-1}. Using the determined values and equation (1) results in:

$$M_n = \frac{K_{cal}}{K_{meas}} \qquad (1)$$

$$M_n = \frac{1228.92 \text{ kg mol}^{-1}}{0.0682 \text{ kg g}^{-1}} \approx 18000 \text{ g mol}^{-1} .$$

With a molecular weight of around 1340 g mol^{-1} per repeating unit (linker **1** + Ru(bda)), this results in an average of 12–14 repeating units per oligomer chain.

The same measurement and calculation adapted to **4b** gives:

$$M_\mathrm{n} = \frac{1228.92\ \mathrm{kg\ mol^{-1}}}{0.1824\ \mathrm{kg\ g^{-1}}} \approx\ 6700\ \mathrm{g\ mol^{-1}}\ .$$

Considering the mixed end-groups within this oligomer, 5 repeating units can be assumed from the VPO experiments.

Molecular Modelling

Based on the average molecular weight and oligomer size obtained from VPO measurements, molecular models of the respective oligomers were constructed to determine the average chain length of the materials.[S9] The geometry-optimized structures after force-field calculations are shown in Figure S7 (**4a**) and Figure S8 (**4b**).

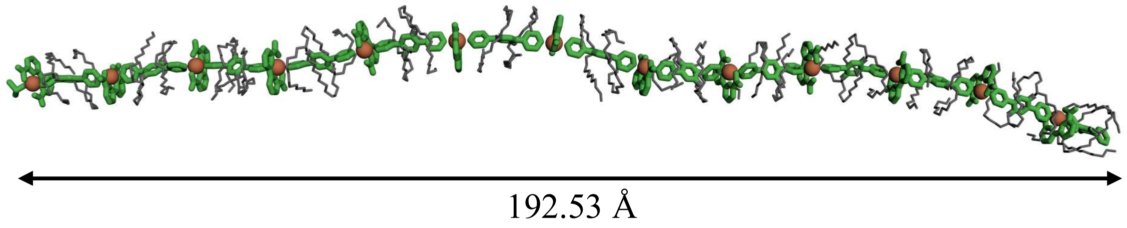

Figure S7. Molecular model of **4a** after force field optimization (green: Linker and bda, grey, oligo (ethyleneglycol) chains, brown: ruthenium).

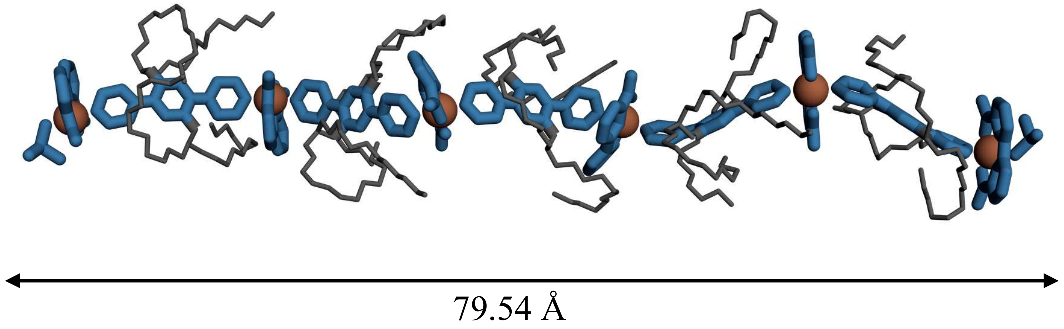

Figure S8. Molecular model of **4b** after force-field optimization (blue: Linker, bda and dmso, grey, oligo (ethyleneglycol) chains, brown: ruthenium).

Diffusion-ordered NMR spectroscopy

DOSY measurements were carried out to determine diffusion coefficients for Ru(bda) oligomers and to approximate the size distribution according to the log-normal model.[S7–S8]

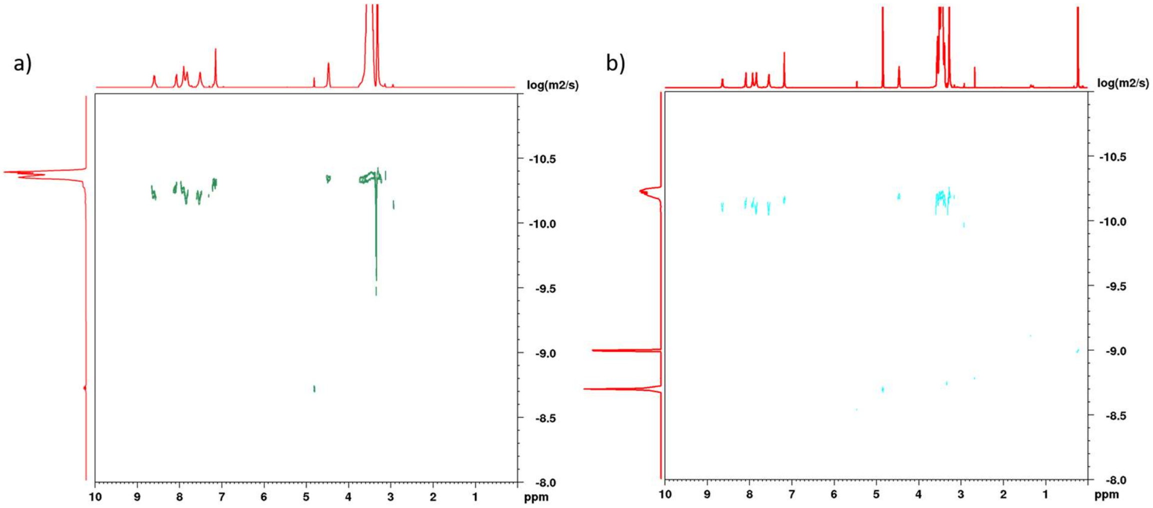

Figure S9. 2D plot of DOSY spectra (CD_2Cl_2/CD_3OD, *v:v*, 1:5, Δ = 100 ms, 295 K) for a) **4a** and b) **4b**.

The log-normal model for DOSY evaluation was chosen due to the polydisperse nature of the oligomer samples. Hereby, the individual signal decay curves are fitted according to equation (2) where the probability for each diffusion coefficient is included by the P_{LN}d) function:

$$\frac{I_g}{I_{g=0}} = \int_0^\infty P(D) \exp\left[-(\gamma\delta g_z)^2 D \left(\Delta - \frac{\delta}{3}\right)\right] dD \tag{2}$$

with $$P_{LN}(D) = \frac{1}{D\sigma_{LN}\sqrt{2\pi}} * \exp\left[-\frac{(\log D - \ln D_0)^2}{2\sigma_{LN}^2}\right] .$$

In this equation, γ is the gyromagnetic ratio ($\gamma(^1H) = 4257.64$ Hz G^{-1}), δ the gradient pulse duration, g_z the gradient strength, D the diffusion coefficient and Δ the diffusion time. σ_{LN} and D_0 are coefficients that determine the shape of the distribution.

Four individual fits were performed as shown in Figure S10a–d for **4a** and Figure S10e–h for **4b**. Table S3 and Table S4 summarize the data obtained from the fits and gives the average value for the parameters needed to define the log-normal distribution for **4a** and **4b**, respectively. Important values that can be derived from the distribution coefficients σ and D_0 are the coefficients D_{mean}, D_{median} and D_{mode} according to equations (3)–(5):

$$D_{mean} = D_0 * \exp\left(\frac{\sigma^2}{2}\right) \tag{3}$$

$$D_{median} = D_0 \tag{4}$$

$$D_{mode} = D_0 * \exp(-\sigma^2) \tag{5}$$

These values are summarized in Table S3 and Table S4 for the individual distributions of the measurements for **4a** and **4b**, respectively.

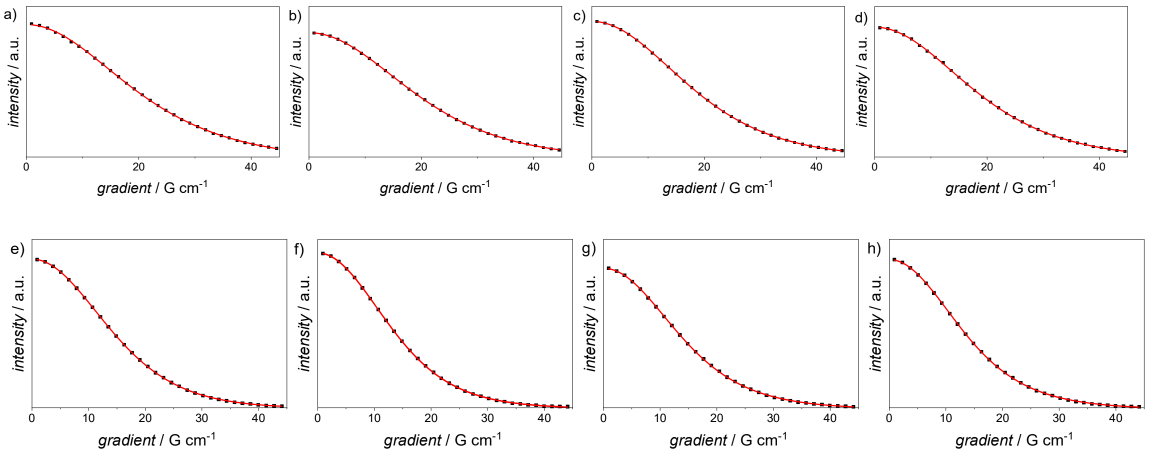

Figure S10. Fit of the individual signal decay curves obtained during DOSY measurement of the signals at a) 4.59 ppm, b) 7.27 ppm, c) 8.02 ppm and d) 8.82 ppm for **4a** and e) 7.15 ppm, f) 7.52 ppm, g) 8.07 ppm and h) 8.62 ppm for **4b**. The black squares shows the measured intensity and the red line the corresponding fit according to the log-normal model.

Table S3. Summary of the σ and D_0 values for **4a** obtained by fitting the signal decay curves shown in Figure S10a–d and the derived parameters D_{mean}, D_{median} and D_{mode}.

Peak / ppm	σ	D_0 / 10^{-11} m^2 s^{-1}	D_{mean} / 10^{-11} m^2 s^{-1}	D_{median} / 10^{-11} m^2 s^{-1}	D_{mode} / 10^{-11} m^2 s^{-1}
4.59	0.7219	4.9634	6.4407	4.9634	2.9476
7.27	0.6079	5.2229	6.2829	5.2229	3.6094
8.02	0.6104	6.0675	7.3099	6.0675	4.1803
8.82	0.6581	5.9716	7.4154	5.9716	3.8725
Average	0.6496	5.5563	6.8622	5.5563	3.6524

Table S4. Summary of the σ and D_0 values for **4b** obtained by fitting the signal decay curves shown in Figure S10e–h and the derived parameters D_{mean}, D_{median} and D_{mode}.

Peak / ppm	σ	D_0 / 10^{-11} m^2 s^{-1}	D_{mean} / 10^{-11} m^2 s^{-1}	D_{median} / 10^{-11} m^2 s^{-1}	D_{mode} / 10^{-11} m^2 s^{-1}
7.15	0.5395	7.4100	8.5708	7.4100	5.5388
7.52	0.6016	9.4165	11.284	9.4165	6.5571
8.07	0.6596	8.5048	10.572	8.5048	5.5045
8.62	0.7339	10.159	13.299	10.159	5.9284
Average	0.6337	8.8726	10.846	8.8726	5.9364

The individual parameters as well as the average parameters can be used to show the distribution of diffusion coefficients as done in Figure S11. The dashed lines correspond to the individual fits while the solid line with filled area marks the result of the average fit which is also shown in Figure 1 of the main text. The conversion of the diffusion coefficient to the hydrodynamic radius is done according to Stokes-Einstein relation (6). This simplified model was used because of lack of knowledge of the oligomers actual shape in the solution phase. For simplified illustration of the length differences between both oligomers, the hydrodynamic diameter was chosen neglecting the shape anisotropy of the molecules.

$$D = \frac{k_B * T}{6\pi * \eta * r_H} \quad . \tag{6}$$

Here, k_B is Boltzmann's constant ($1.38064852 \times 10^{-23}$ m^2 kg s^{-2} K^{-1}), T is the absolute temperature, η the solution viscosity and r_H the hydrodynamic radius. The size was calculated under the assumption that the solution viscosity of the solvent mixture is approximately equal to the viscosity of pure deuterated methanol ($\eta(CD_3OD) = 0.60 \times 10^{-3}$ kg m^{-1} s^{-1}).[S10]

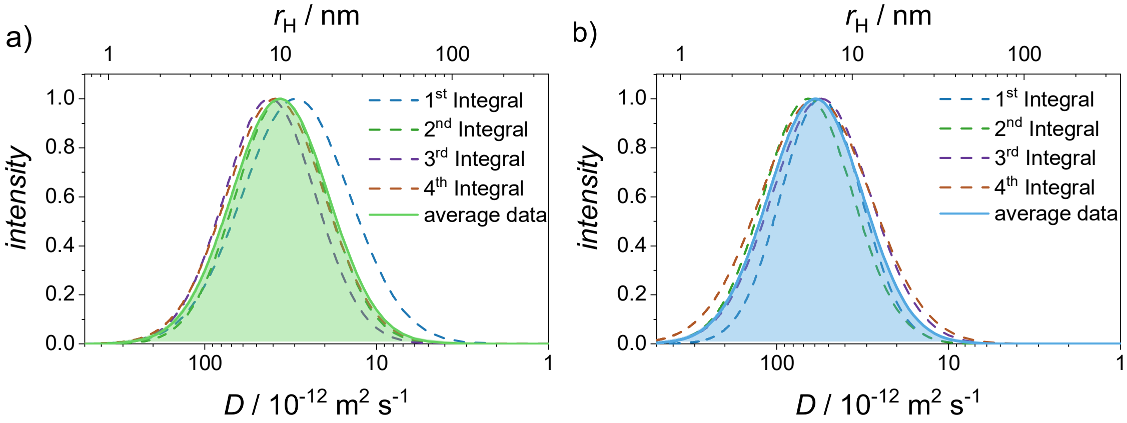

Figure S11. Size distribution of a) **4a** and b) **4b** as determined by DOSY measurements. The dashed lines show the individual results for four different proton signals that were fitted according to the log-normal model. The solid line with filled area shows the distribution of the average of the four signals.

Atomic force microscopy

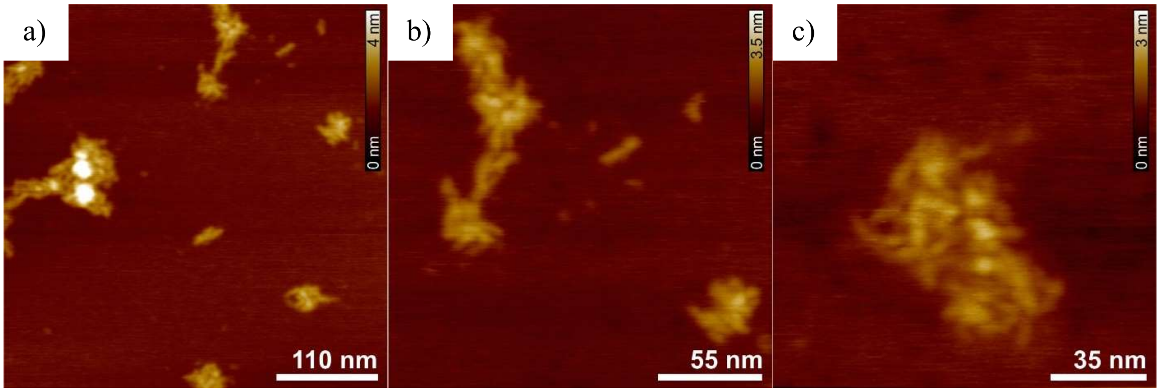

Figure S12. AFM height images obtained after spin-coating of a solution of **4a** in water ($c_{Ru} = 1 \times 10^{-5}$ M) on mica substrate. The Z-scale is a) 4.0 nm and b) 3.5 nm and c) 3.0 nm and the scale bar is a) 110 nm b) 55 nm and c) 35 nm, respectively.

The AFM images after spin-coating a sample of **4a** in pure water ($c_{Ru} = 1 \times 10^{-5}$ M) on a mica substrate show rod-like structures with a height of 0.60 ± 0.1 nm corresponding to the axis perpendicular to the oligomer long axis. The images show a pronounced tendency for flake-like agglomerated structures and barely any individual fibers are observed. This fact shows the enhanced tendency for extended oligomeric structures to form conglomerates under aqueous conditions.

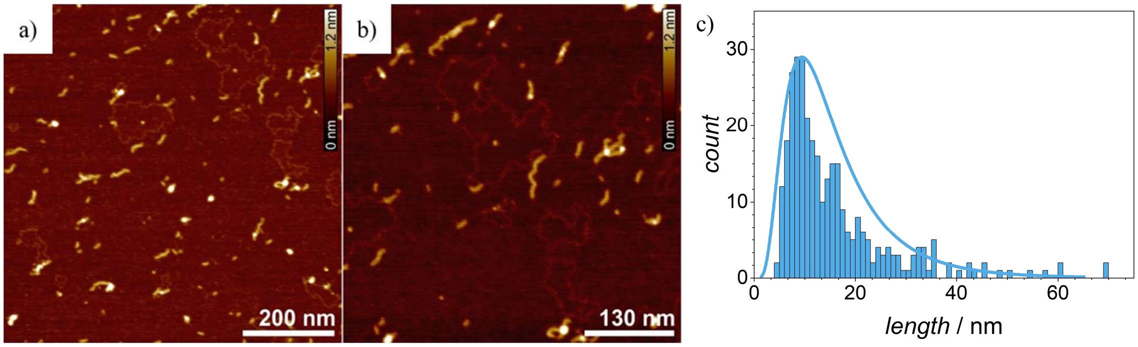

Figure S13. AFM height images obtained after spin-coating of an aqueous solution of **4b** ($c = 1 \times 10^{-5}$ M) on mica substrate. The Z-scale is 1.2 nm and the scale bar is a) 200 nm and b) 130 nm, respectively. c) Histogram of the oligomer length distribution obtained by visual analysis of the oligomer lengths in the AFM image.

The length of individual oligomers of **4b** was measured visually for AFM images obtained after spin-coating an aqueous solution of **4b** on mica substrate. The observed, fiber-like structures have a height of around 0.5 ± 0.5 nm corresponding to the axis perpendicular to the oligomer long axis. The length of the structures shows a great disparity between around 7 nm and up to 60 nm. Via statistical analysis, a log-normal distribution is obtained (Figure S13c), which is in good agreement with the DOSY NMR and VPO data. The fact that from DOSY data a slightly

larger size of the oligomers was estimated can on the one hand be rationalized by the shape anisotropy of the oligomeric structures. On the other hand, the number average molecular weight was determined by VPO and AFM while for DOSY a mass averaged distribution is obtained.

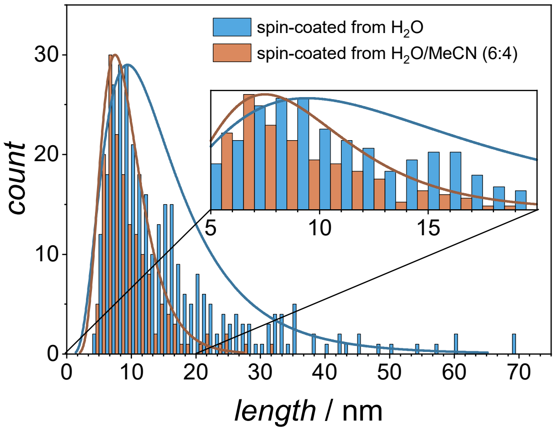

Figure S14. Comparison of the length distribution for **4b** after spin-coating from either a water/acetonitrile mixture (*v*:*v*, 6:4, $c = 1 \times 10^{-5}$ M, brown bars) or pure water ($c = 1 \times 10^{-5}$ M, blue bars). The curves correspond to the respective lognormal fit of the histogram.

Spin-coating of **4b** from pure water and 40 % acetonitrile in water showed different length distributions (Figure S14). As the structures obtained from pure water are slightly larger, the formation of self-assembled structures is proposed for pure water or mixtures with low acetonitrile content. The fact that the self-assemblies are small contributes to the high solubility of **4b** under aqueous conditions.

Summary

Table S5. Overview over various analytical techniques for the determination of average oligomer lengths.

	**Technique**	**Amount of repeating units**	**Length determination / nm**	**Corresponding Molecular weight**
**4a**	CHN	16 – 17	24 – 26	M_w
	DOSY	13 – 14	19 – 20	M_w
	NMR	17 – 18	26 – 28	M_n
	AFM	10 – 14	15 – 22	M_n
	VPO	12 – 14	18 – 21	M_n
**4b**	CHN	6 – 7	9 – 10	M_w
	DOSY	7 – 8	12 – 13	M_w
	NMR	6 – 7	9 – 10	M_n
	AFM	5 – 6	8 – 9	M_n
	VPO	5	7.9	M_n

Optical and electrochemical properties

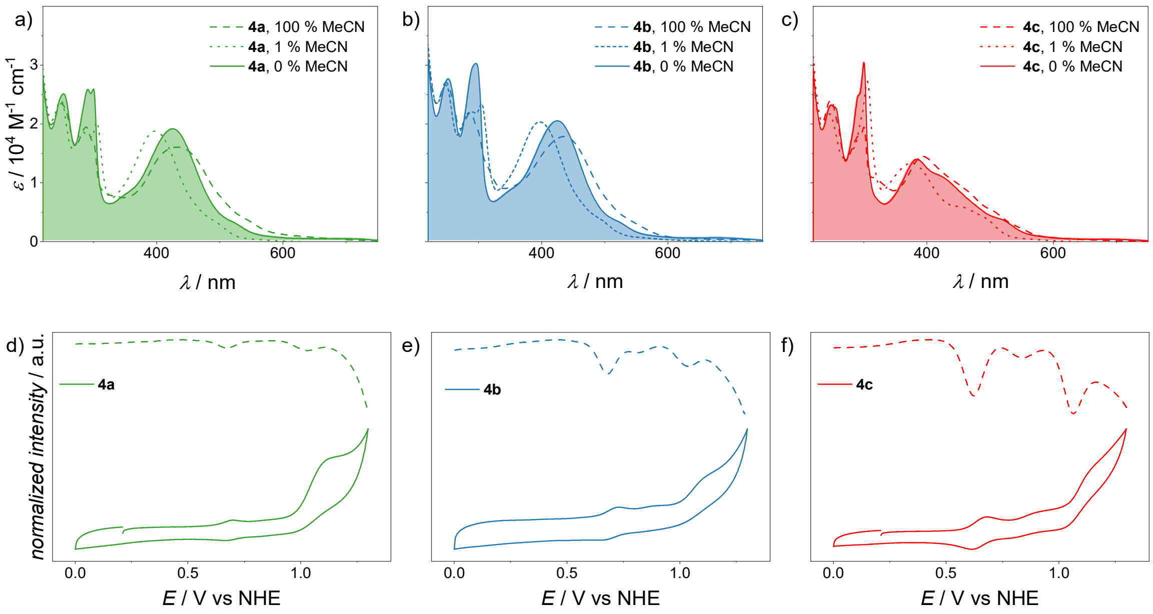

Figure S15. UV/vis absorption spectra of a) **4a**, b) **4b** and c) **4c** ($c_{\mathrm{Ru}} = 2.0 \times 10^{-5}$ M) in varying mixtures of water and acetonitrile (dashed line: 100 % MeCN, dotted line: 1 % MeCN, solid line: 0 % MeCN). Cyclic voltammetry and differential pulse voltammetry of d) **4a**, e) **4b** and f) **4c** ($c_{\mathrm{Ru}} = 2.00 \times 10^{-4}$ M) in aqueous phosphate buffer (pH = 7.0, c = 0.1 M).

Visible light-driven water oxidation catalysis

Lamp spectrum

The recorded lamp spectrum of the 150 W xenon arc lamp equipped with a solar filter is shown in Figure S16.

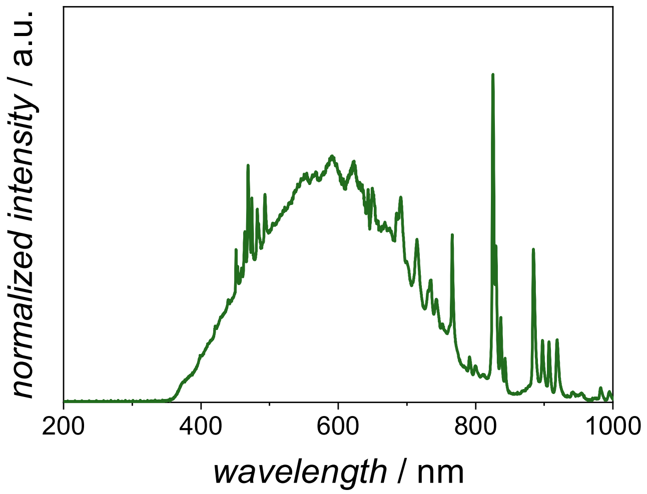

Figure S16. Lamp spectrum of the 150 W xenon arc lamp used as external light source during visible light-driven water oxidation catalysis. The light is passed through a solar filter before sample illumination.

Mechanisms of molecular water oxidation catalysts

The detailed discussion of the two most prominent reaction mechanisms during water oxidation in molecular water oxidation catalysts can be found in detail in the literature.[S11] After generation of a high valent metal oxo species, the rate determining step can proceed via one of two pathways. The oxygen evolution in the Ru(bda) family usually proceeds via the interaction of two metal oxo radicals (I2M) mechanism. Hereby, two of the high valent metal oxo species react together to form a peroxo intermediate before oxygen evolution. The other pathway discussed in literature is based on the nucleophilic attack of a water molecule on the metal oxo complex forming a hydroperoxo intermediate before oxygen evolution. There are two distinct features that allow differentiation between the two mechanisms by experiments. On one hand, the I2M mechanism exhibits second-order kinetics with respect to catalyst concentration while in the WNA mechanism shows first-order kinetics. Additionally, WNA mechanism includes a proton coupled electron transfer in the rate determining step which is why strong H/D kinetic isotope effects can be expected on the rate of oxygen evolution if this pathway is occurring.

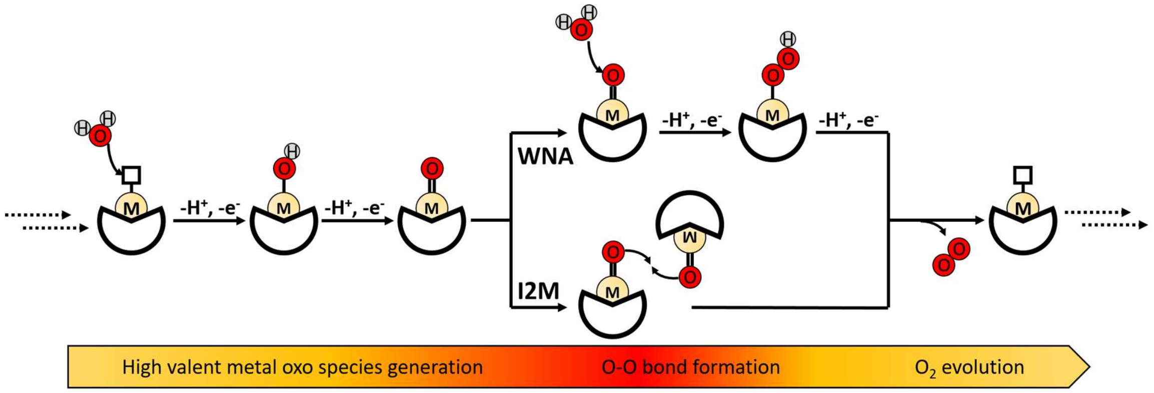

Figure S17. Schematic illustration of the water oxidation mechanisms in molecular water oxidation catalysts. The rate determining steps are indicated for the WNA (water nucleophilic attack) and I2M (Interaction of two metal-oxo radicals) mechanisms.

Solvent-dependent water oxidation catalysis

For solvent-dependent measurements, water oxidation catalysis with constant amounts of photosensitizer (Ru(deeb)$_2$(bpy)Cl$_2$, $c = 0.2$ mM) and sacrificial electron acceptor ($Na_2S_2O_8$, $c = 37.5$ mM) at 293 K was performed in varying ratios of 50 mM phosphate buffer (p$H = 7.2$) and acetonitrile. The irradiation power of a 150 W xenon lamp as external light source was calibrated to 100 mW cm^{-2} (one sun at AM1.5G).

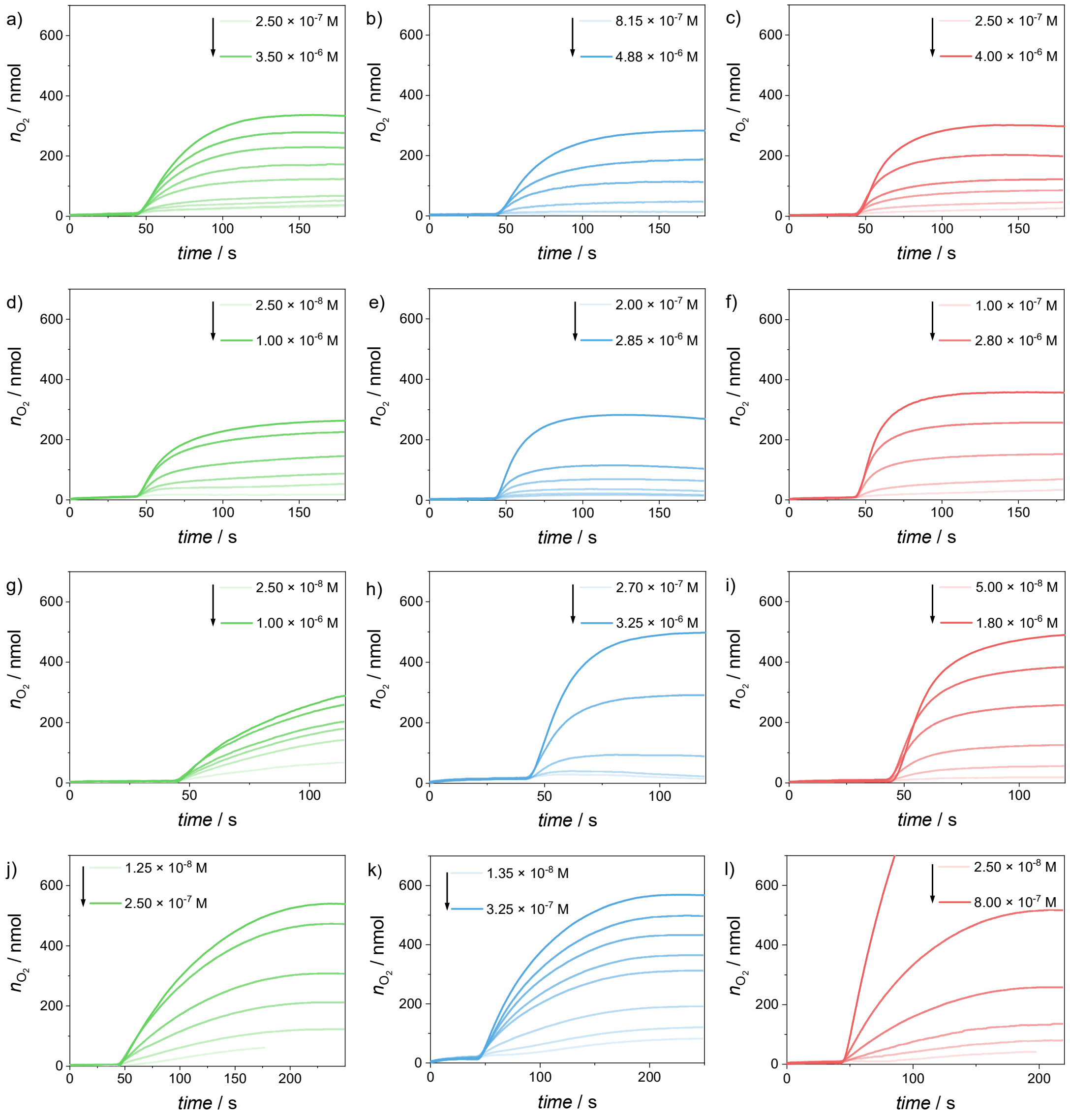

Figure S18. Oxygen evolution over time during visible light-driven water oxidation catalysis with varying concentrations of a), d), g), j) **4a**, b), e), h), k) **4b** and c), f), i), l) **4c**. The amount of photosensitizer (Ru(deeb)$_2$(bpy)Cl$_2$, $c = 0.2 \times 10^{-3}$ M) and sacrificial electron acceptor ($Na_2S_2O_8$, $c = 37.5 \times 10^{-3}$ M) were kept constant during the experiment and irradiation was started after 40 s. The experiments were carried out in aqueous phosphate buffer (pH = 7.2) containing a)–c) 20 %, d)–f) 10 %, g)–i) 1 % and j)–l) 0 % acetonitrile as co-solvent.

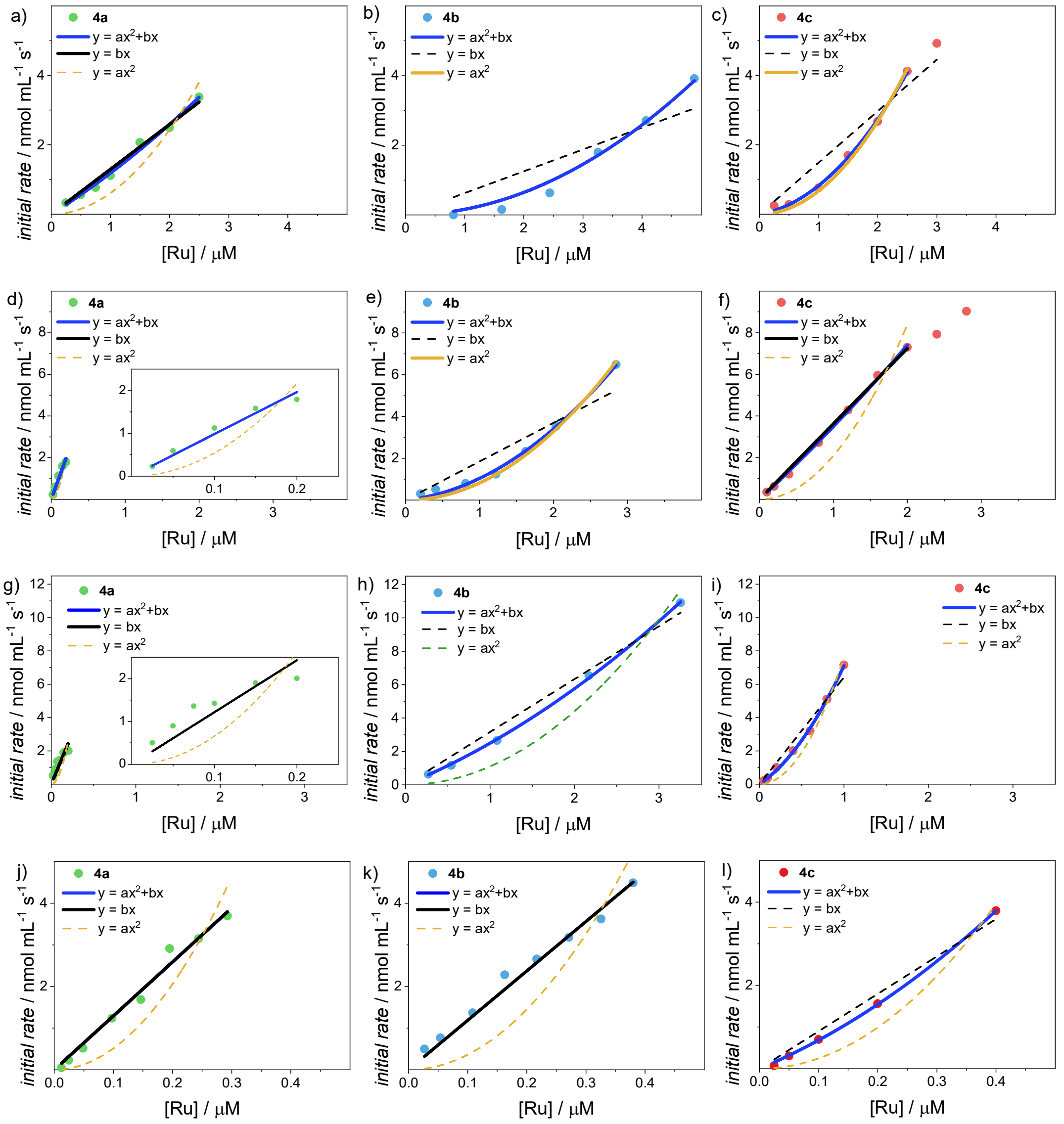

Figure S19. Plots of the initial rates of oxygen evolution (determined by linear regression during the first few seconds of catalysis) for a), d), g), j) **4a** (green dots), b), e), h), k) **4b** (blue dots) and c), f), i), l) **4c** (red dots) in aqueous phosphate buffer containing a)–c) 20 %, d)–f) 10 %, g)–i) 1 % and j)–l) 0 % acetonitrile as co-solvent. All data sets were fitted according to three different kinetic models and fit parameters are summarized in Table S6. The fits with the highest R^2 value are indicated using solid lines while fits with lower R^2 value are dashed. Fit parameters a and b were restricted to positive values.

Table S6. Summary of fit parameters for all water oxidation experiments using different kinetic models. Parameters a and b are restricted to positive values during fitting. For all measurements, the most accurate kinetic model is marked in color.

Compound	Acetonitrile content / %	$y = ax^2$		$y = ax^2 + bx$			$y = bx$	
		a	R^2	a	b	R^2	b	R^2
**4a**	**20**	0.3043	0.8256	0.0547	1.0752	0.9970	**1.2924**	**0.9946**
	**10**	27.146	0.5032	0.0000	9.8294	0.9582	**9.8294**	**0.9902**
	**1**	32.863	–0.7223	0.0000	12.162	0.9564	**12.162**	**0.9564**
	**0**	25.726	0.8208	0.1473	12.878	0.9937	**12.948**	**0.9937**
**4b**	**20**	**0.0809**	**0.9831**	0.0809	0.0000	0.9831	0.6266	0.9001
	**10**	0.4091	0.9881	**0.3329**	**0.3690**	**0.9976**	1.8458	0.9422
	**1**	0.5514	0.9300	**0.1936**	**2.1068**	**0.9998**	3.1675	0.9915
	**0**	18.140	0.6355	0.0000	11.867	0.9958	**11.867**	**0.9958**
**4c**	**20**	**0.3354**	**0.9913**	0.2702	0.2834	0.9989	1.4852	0.9708
	**10**	1.0489	0.8610	**0.1030**	**3.2907**	**0.9995**	3.6261	0.9989
	**1**	3.8091	0.9582	**1.8539**	**3.4011**	**0.9993**	6.4195	0.9844
	**0**	12.389	0.9322	**4.4065**	**5.9604**	**0.9995**	8.9849	0.9897

Table S7. Summary of TOF_{max} and TON_{max} values for visible light-driven water oxidation catalysis with **4a–c**. The experiments were carried out with $Ru(deeb)_2(bpy)Cl_2$ (c = 0.2 mM) as photosensitizer, $Na_2S_2O_8$ (c = 37.5 mM) as sacrificial electron acceptor and various mixtures of 50 mM aqueous phosphate buffer (pH = 7.2) and acetonitrile as solvent.

Compound	Acetonitrile content / %	TOF_{max} / s^{-1} (c_{Ru} / M)	TON (c_{Ru} / M)
**4a**	20	1.38 (1.50 × 10^{-6})	46 (3.00 × 10^{-6})
	10	10.6 (1.50 × 10^{-7})	190 (1.50 × 10^{-7})
	1	12.7 (1.50 × 10^{-7})	590 (1.50 × 10^{-7})
	0	14.9 (2.00 × 10^{-7})	1200 (2.00 × 10^{-7})
**4b**	20	0.80 (4.90 × 10^{-6})	28 (4.90 × 10^{-6})
	10	2.27 (2.90 × 10^{-6})	50 (2.90 × 10^{-6})
	1	3.35 (3.30 × 10^{-6})	75 (3.30 × 10^{-6})
	0	14.0 (1.60 × 10^{-7})	1070 (2.50 × 10^{-8})
**4c**	20	1.65 (2.50 × 10^{-6})	35 (2.50 × 10^{-6})
	10	3.72 (1.60 × 10^{-6})	63 (2.00 × 10^{-6})
	1	7.24 (1.40 × 10^{-6})	136 (1.40 × 10^{-6})
	0	9.48 (4.00 × 10^{-7})	640 (2.00 × 10^{-7})

Determination of the reaction order

To determine the reaction order during visible light-driven water oxidation catalysis, the plot of –log($dn(O_2)/dt$) against –log(c_{Ru}) was fitted with a linear equation giving an indication of the reaction order observed during catalysis.

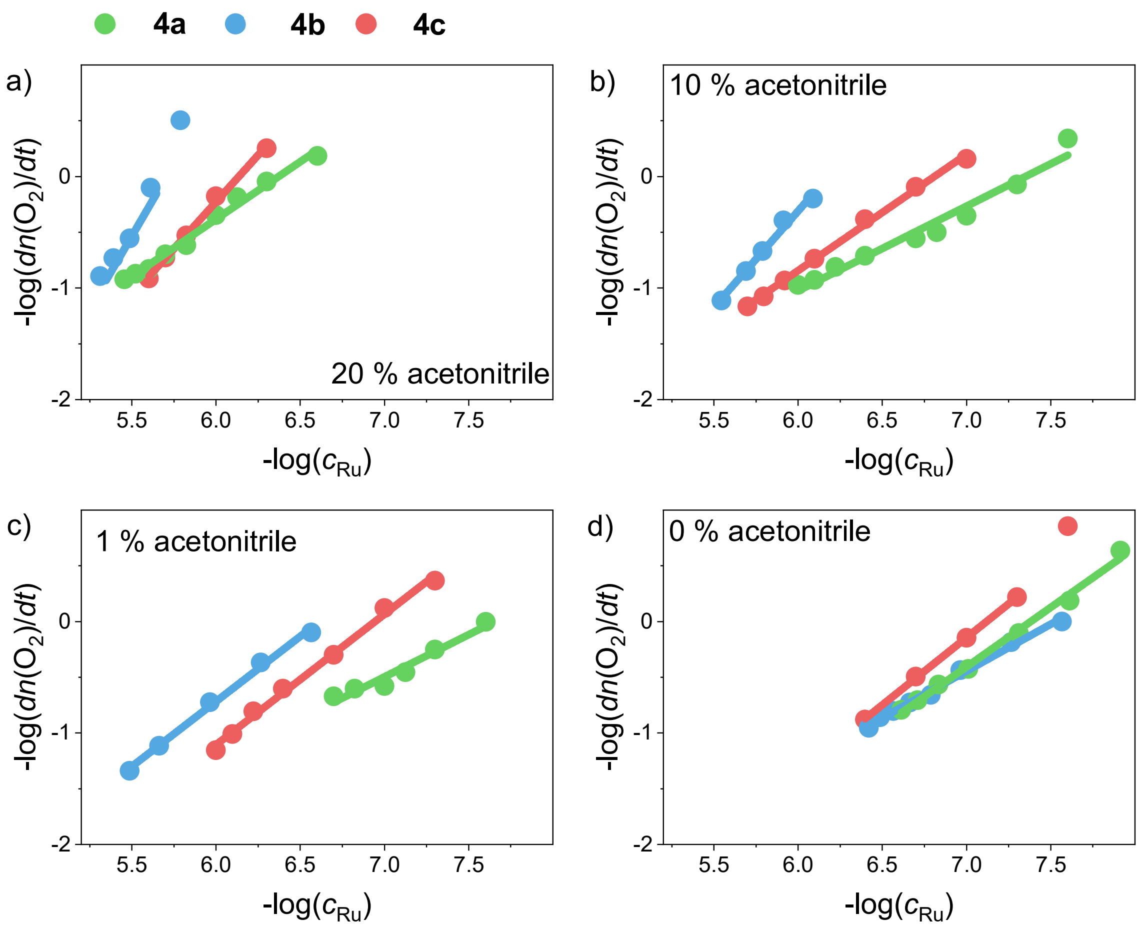

Figure S20. –log plot of the initial rate of oxygen evolution over the concentrations of ruthenium centers for **4a** (green dots), **4b** (blue dots) and **4c** (red dots) for the experiments carried out in aqueous phosphate buffer containing a) 20 %, b) 10 %, c) 1 % and d) 0 % acetonitrile as organic co-solvent. The straight lines in the graphs represent the fits used in determination of the reaction order.

Kinetic isotope effect measurements

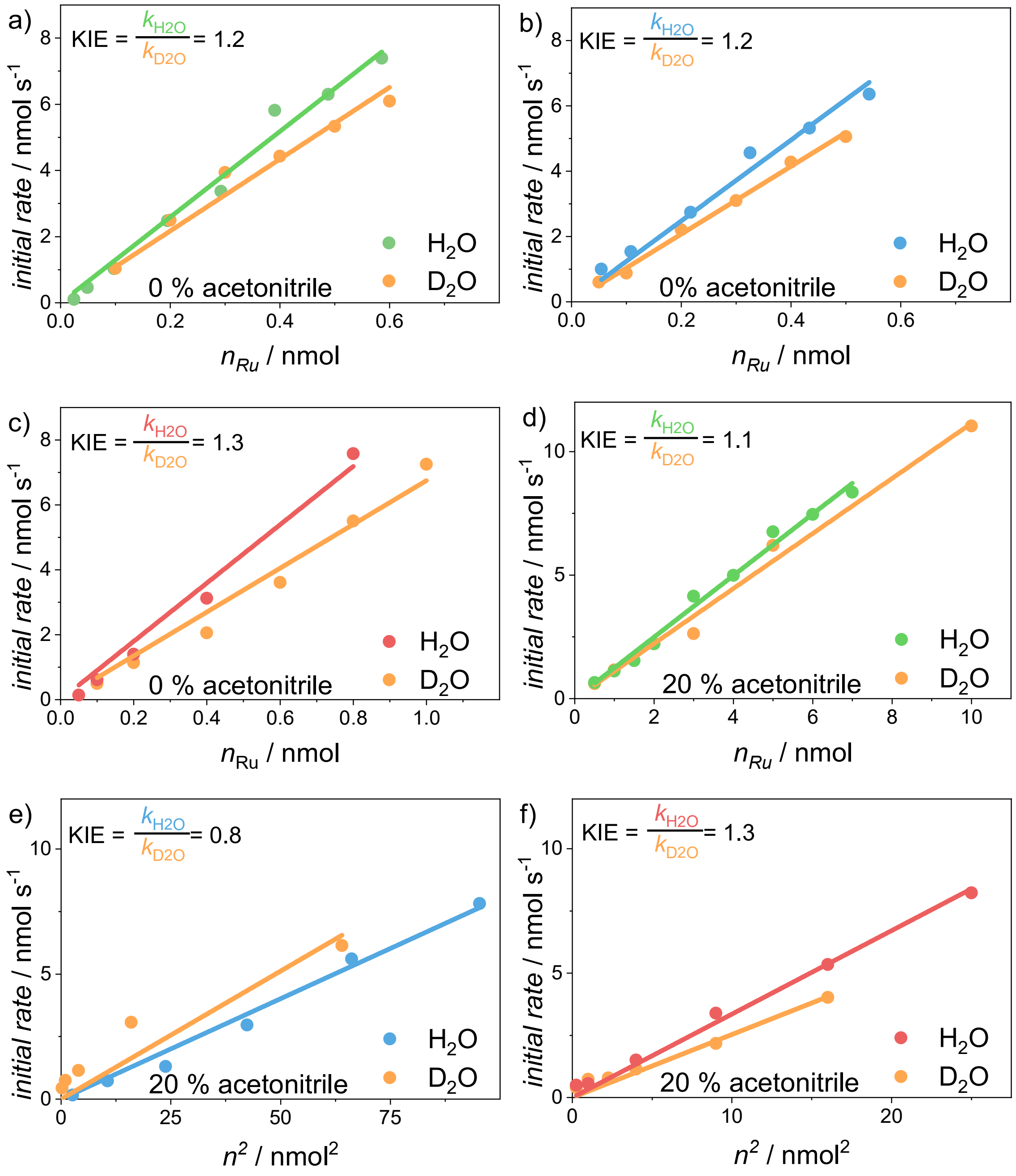

Figure S21. Summary of initial rates of oxygen evolution against the amount of ruthenium centers in mixtures of phosphate buffered (p*H*(*D*) = 7.2, $c = 50 \times 10^{-3}$ M) H_2O (**4a**: green, **4b**: blue, **4c**: red) or D_2O (orange) with a)–c) 0 % acetonitrile and d)–f) 20 % acetonitrile as organic co-solvent. The amount of photosensitizer ($Ru(deeb)_2(bpy)Cl_2$, $c = 0.2 \times 10^{-3}$ M) and sacrificial electron acceptor ($Na_2S_2O_8$, $c = 37.5 \times 10^{-3}$ M) was kept constant throughout the experiments and irradiation was started after 40 s. The linear regression is used for the determination of individual reaction rates $k(H_2O)$ and $k(D_2O)$ for kinetic isotope effect experiments.

Table S8. Summary of data obtained from KIE experiments during visible light-driven water oxidation catalysis of **4a–c** with $Ru(deeb)_2(bpy)Cl_2$ ($c = 0.2 \times 10^{-3}$ M) as photosensitizer and $Na_2S_2O_8$ as sacrificial electron acceptor ($Na_2S_2O_8$, $c = 37.5 \times 10^{-3}$ M).

Compound	Acetonitrile content / %	k_{H2O}	k_{D2O}	KIE
**4a**	0	12.9 ± 1.67 s^{-1}	10.9 ±1.10 s^{-1}	1.2 ± 0.17
	20	1.25 ± 0.11 s^{-1}	1.11 ± 0.12 s^{-1}	1.1 ± 0.21
**4b**	0	11.9 ± 1.07 s^{-1}	10.4 ± 0.97 s^{-1}	1.1 ± 0.19
	20	0.080 ± 0.01 nmol^{-1} s^{-1}	0.102 ± 0.25 nmol^{-1} s^{-1}	0.8 ± 0.27
**4c**	0	8.98 ± 1.25 s^{-1}	6.75 ± 0.85 s^{-1}	1.3 ± 0.12
	20	0.335 ± 0.09 nmol^{-1} s^{-1}	0.253 ± 0.04 nmol^{-1} s^{-1}	1.3 ± 0.26

Table S9. Reported data for catalysts tested in visible light-driven water oxidation catalysis as comparison to values obtained for coordination oligomers **4**. If catalyst concentrations are missing, first order WNA mechanism was observed making these values negligible.

	TOF / s^{-1} (c(cat) / nM)	TOF per Ru / s^{-1}	TON (c(cat) / nM)	TON per Ru	Photosensitizer	Solvent / % in water	Reference
**S5**	/	/	8 (50000)[a]	8	$Ru(bpy)_3$	10% MeCN	[S12]
	/	/	129 (1000)[b]	129	$Ru(deeb)_2(bpy)$	/	[S13]
	0.99 (200) [c]	0.99	405 (200)[c]	405	$Ru(deeb)_2(bpy)$	1% MeCN	[S14]
**S6**	13.1[d]	4.4	1250 (86)[d]	417	$Ru(bpy)_3$	50% MeCN	[S15]
	10.9[d]	3.6	430	215	$Ru(bpy)_3$	50% MeCN	[S16]
	2.8[d]	0.9	220	73	$Ru(deeb)_2(bpy)$	50% MeCN	[S17]
**S7**	11[d]	3.7	430	215	$Ru(bpy)_3$	50% MeCN	[S18]
**S8**	9.5[d]	3.2	550	183	$Ru(bpy)_3$	50% MeCN	[S17]
	2.2[d]	0.7	180	60	$Ru(deeb)_2(bpy)$	50% MeCN	[S17]
	2.9[f]	1.0	270	90	$Ru(bpy)_3$	5% MeCN	[S17]
	10.8[f]	3.6	320	107	$Ru(deeb)_2(bpy)$	5% MeCN	[S17]
**S9**	10.8[d]	3.6	480	160	$Ru(bpy)_3$	50% MeCN	[S16]
**S10**	7.8[d]	2.6	380	127	$Ru(bpy)_3$	50% MeCN	[S16]
**S11**	9.1[d]	3.0	400	133	$Ru(bpy)_3$	50% MeCN	[S16]
**S12**	2.0[d]	0.7	170	57	$Ru(bpy)_3$	50% MeCN	[S16]
**S13**	1.7[d]	0.6	120	40	$Ru(bpy)_3$	50% MeCN	[S16]
**S14**	4.8[d]	1.6	260	87	$Ru(bpy)_3$	50% MeCN	[S16]
**S15**	/	/	369 (500)[b]	185	$Ru(deeb)_2(bpy)$	/	[S13]
**S16**	/	/	535 (330)[b]	178	$Ru(deeb)_2(bpy)$	/	[S13]
**S17**	15.5[d]	7.8	460	230	$Ru(bpy)_3$	40% MeCN	[S19]
	13.3[d]	6.7	540	270	$Ru(bpy)_3$	50% MeCN	[S19]
**S18**	1.1[d]	0.6	36	18	$Ru(bpy)_3$	50% MeCN	[S18]
**S19**	23[d]	5.8	500	125	$Ru(bpy)_3$	50% MeCN	[S18]
**S20**	11.1[c]	5.6	935[c]	468	$Ru(deeb)_2(bpy)$	/	[S14]

	TOF / s^{-1} (c(cat) / nM)	TOF per Ru / s^{-1}	TON (c(cat) / nM)	TON per Ru	Photosensitizer	Solvent / % in water	Reference
**S21**	9.2[c]	4.6	2373[c]	1187	$Ru(deeb)_2(bpy)$	/	[S14]
**4a**	14.9 (200)	14.9	1200	1200	$Ru(deeb)_2(bpy)$	/	this work
**4b**	14.0 (160)	14.0	1070	1070	$Ru(deeb)_2(bpy)$	/	this work
**4c**	9.48 (400)	9.48	640	640	$Ru(deeb)_2(bpy)$	/	this work

[a] $c_{PS} = 1.00 \times 10^{-4}$ M, $c_{SEA} = 5.0 \times 10^{-2}$ M.
[b] $c_{PS} = 1.00 \times 10^{-3}$ M, $c_{SEA} = 1.0 \times 10^{-2}$ M.
[c] $c_{PS} = 2.00 \times 10^{-4}$ M, $c_{SEA} = 1.0 \times 10^{-2}$ M.
[d] $c_{PS} = 1.50 \times 10^{-3}$ M, $c_{SEA} = 3.70 \times 10^{-2}$ M.
[e] $c_{PS} = 1.50 \times 10^{-3}$ M, $c_{SEA} = 3.70 \times 10^{-2}$ M.
[f] $c_{PS} = 2.00 \times 10^{-4}$ M, $c_{SEA} = 3.70 \times 10^{-2}$ M.

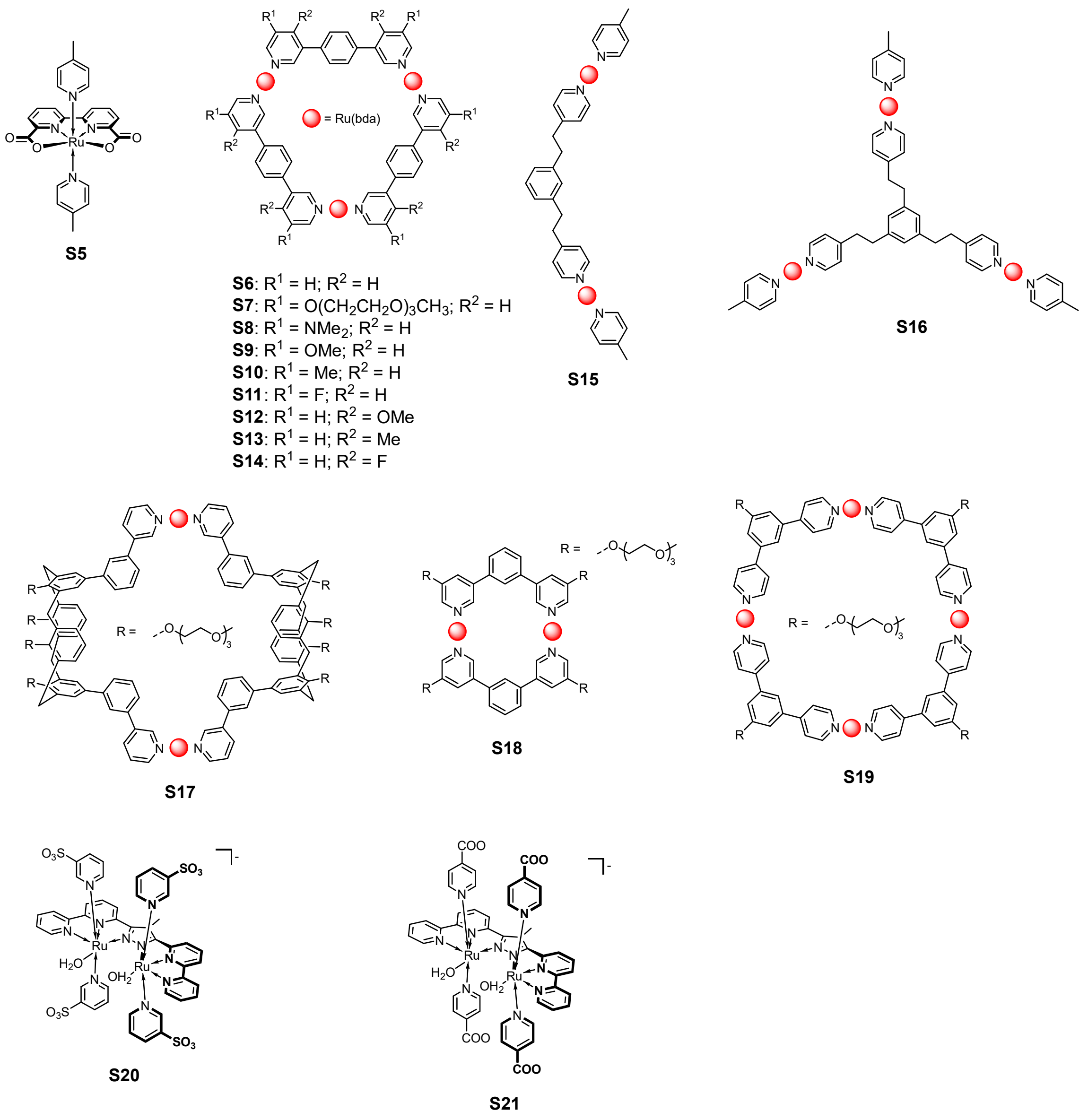

Figure S22. Molecular structures of catalysts **S5–S21** tested in visible light-driven water oxidation catalysis. Obtained data is summarized in Table S9.

Analytical data for molecular precursors and Ru(bda) oligomers

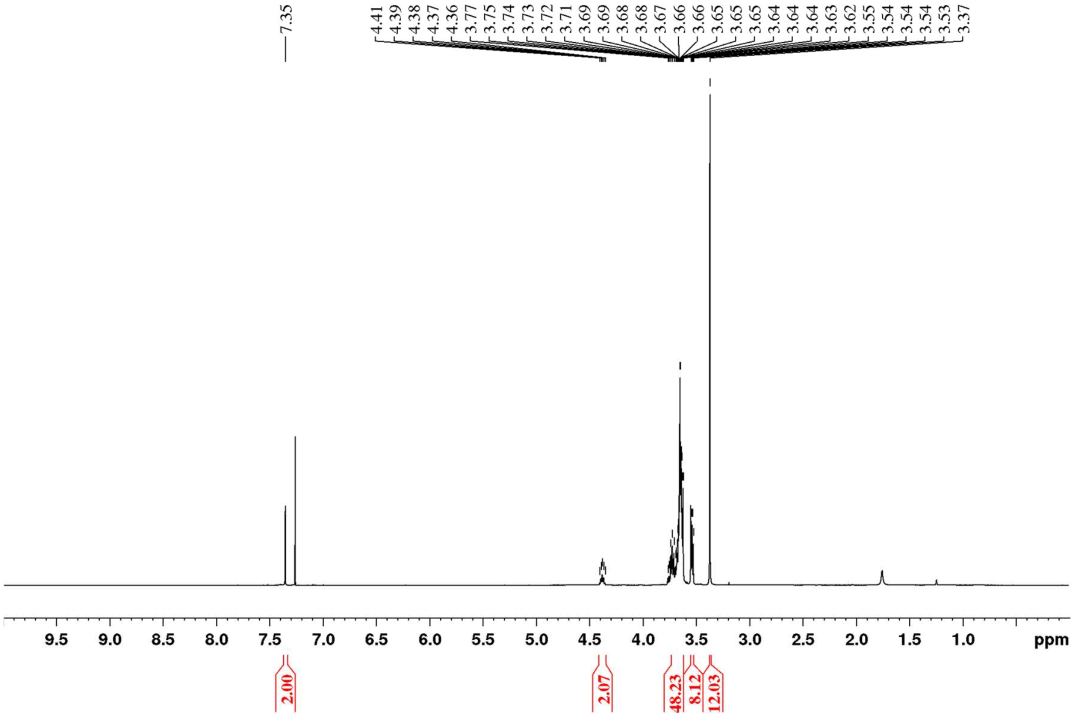

Figure S23. ^{1}H NMR spectrum (400 MHz, $CDCl_3$, 295 K) of **S3**.

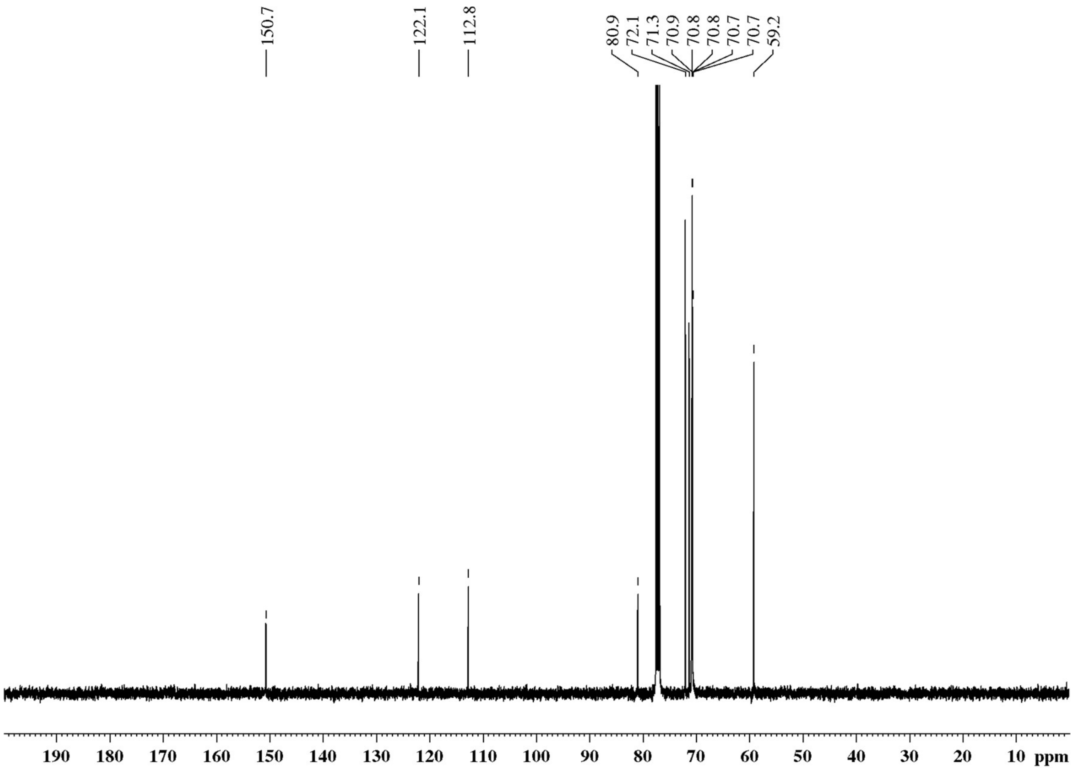

Figure S24. ^{13}C NMR spectrum (400 MHz, $CDCl_3$, 295 K) of **S3**.

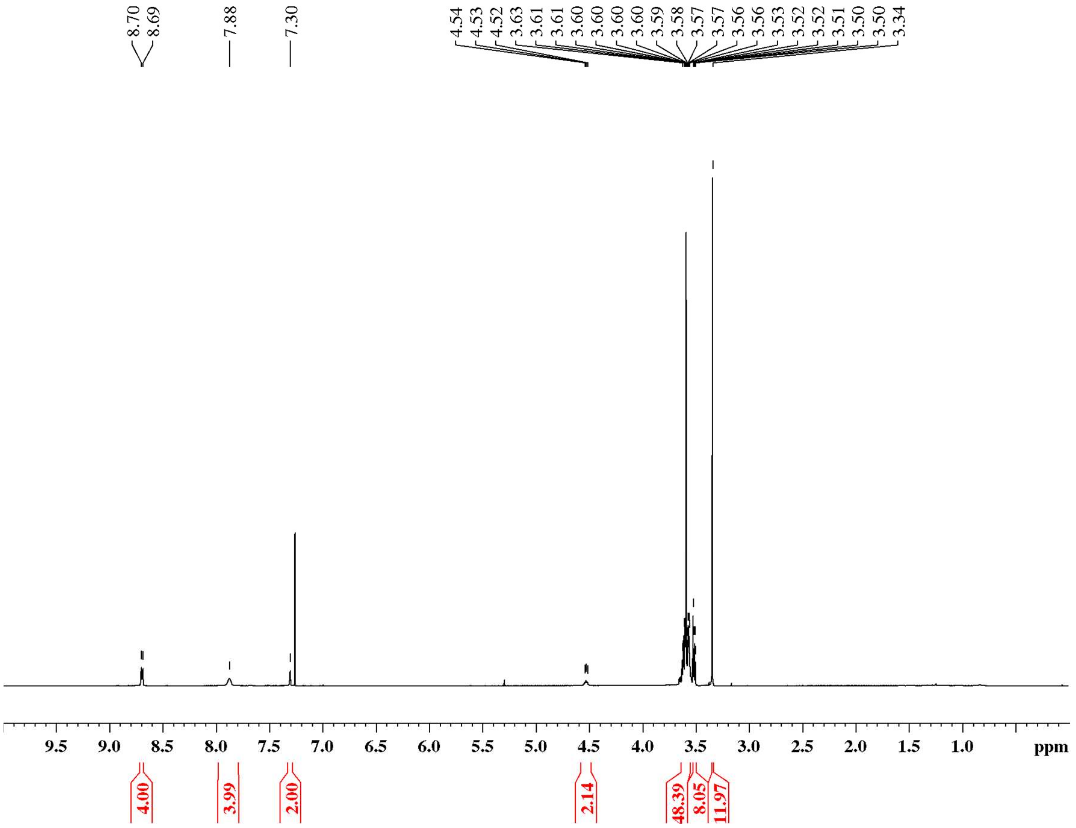

Figure S25. ^{1}H NMR spectrum (400 MHz, $CDCl_3$, 295 K) of **1.**

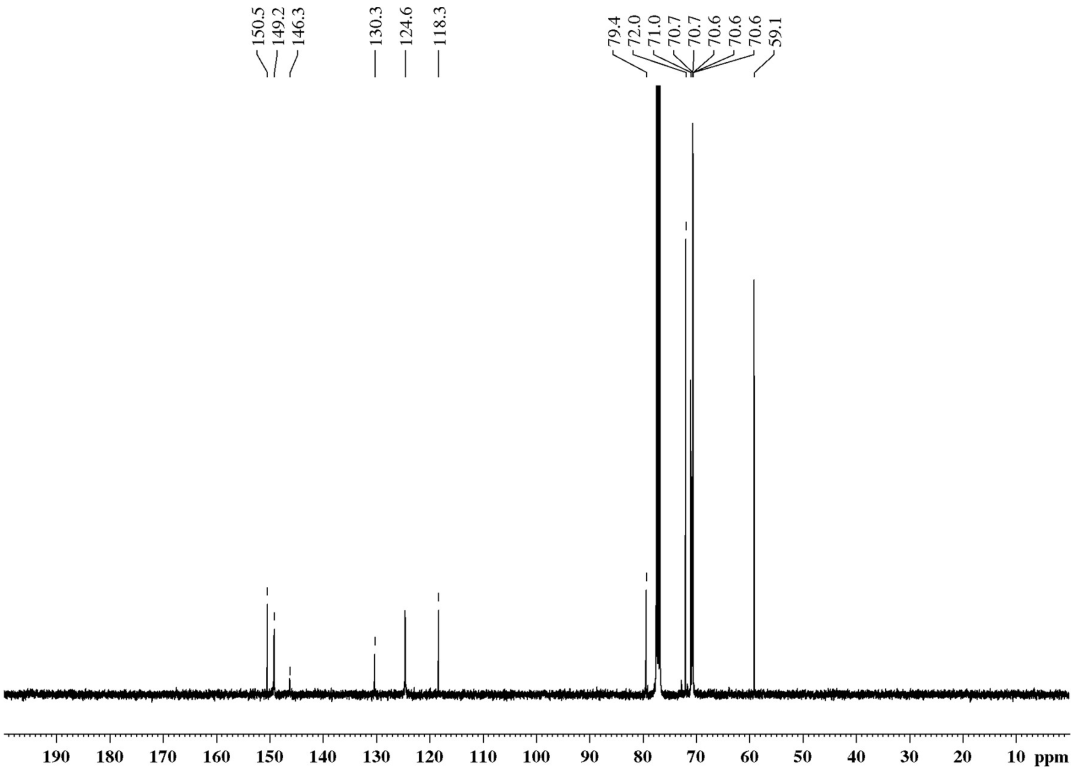

Figure S26. ^{13}C NMR spectrum (400 MHz, $CDCl_3$, 295 K) of **1.**

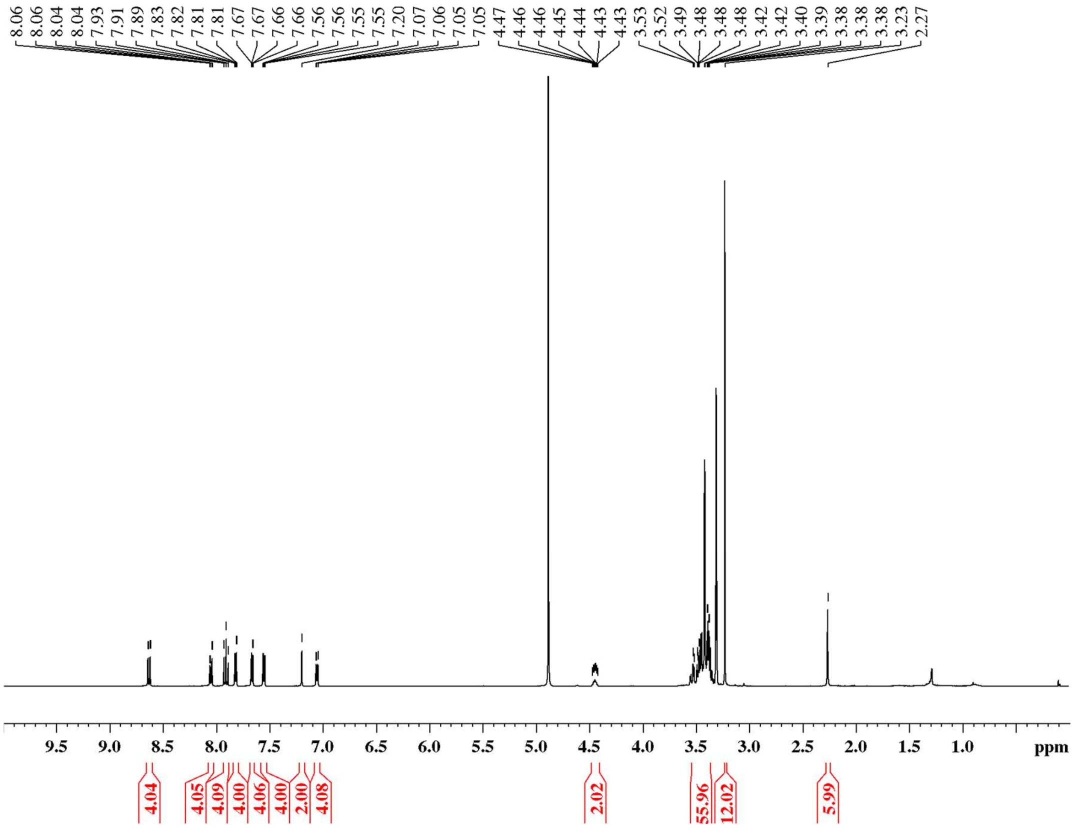

Figure S27. ^{1}H NMR spectrum (400 MHz, $CDCl_3$, 295 K) of **4c**.

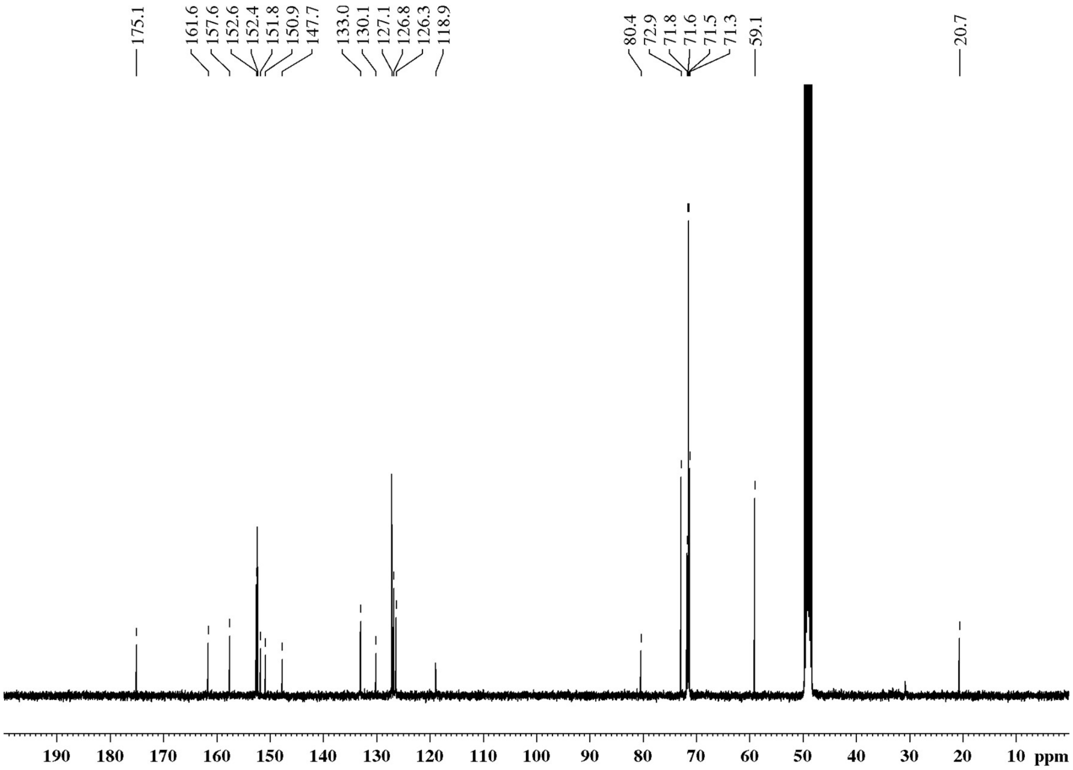

Figure S28. ^{13}C NMR spectrum (100 MHz, CD_3OD, 295 K) of **4c**.

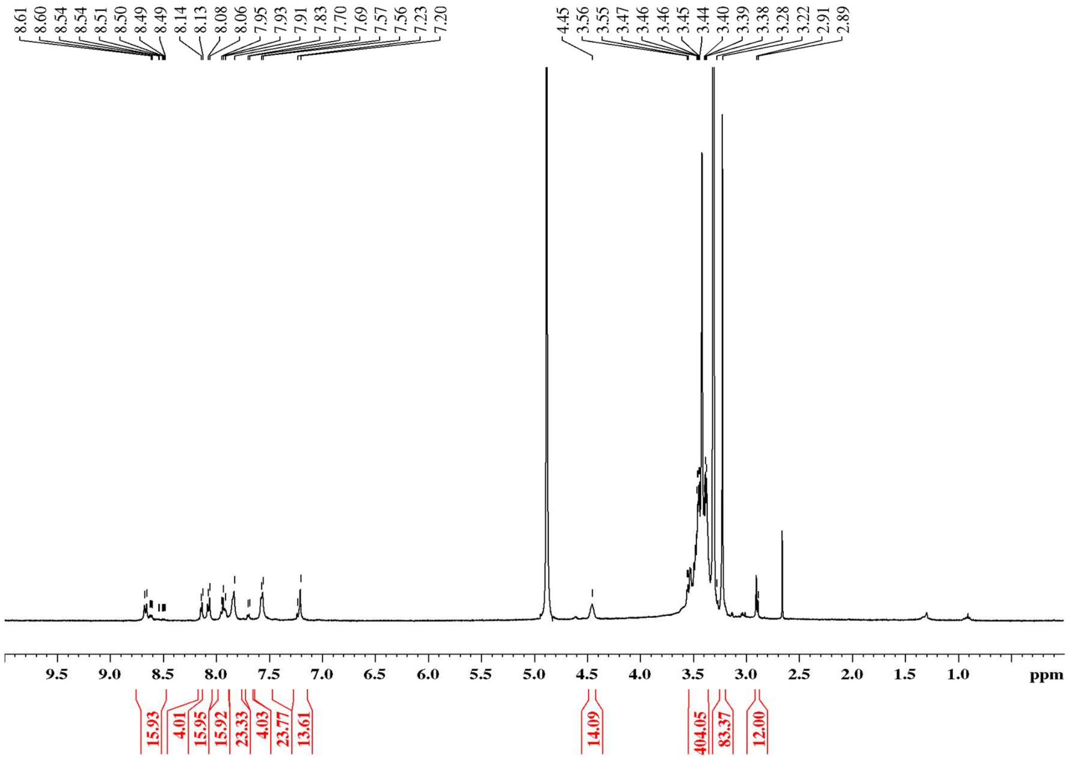

Figure S29. ^{1}H NMR spectrum (400 MHz, CD_3OD, 295 K) of **4b**.

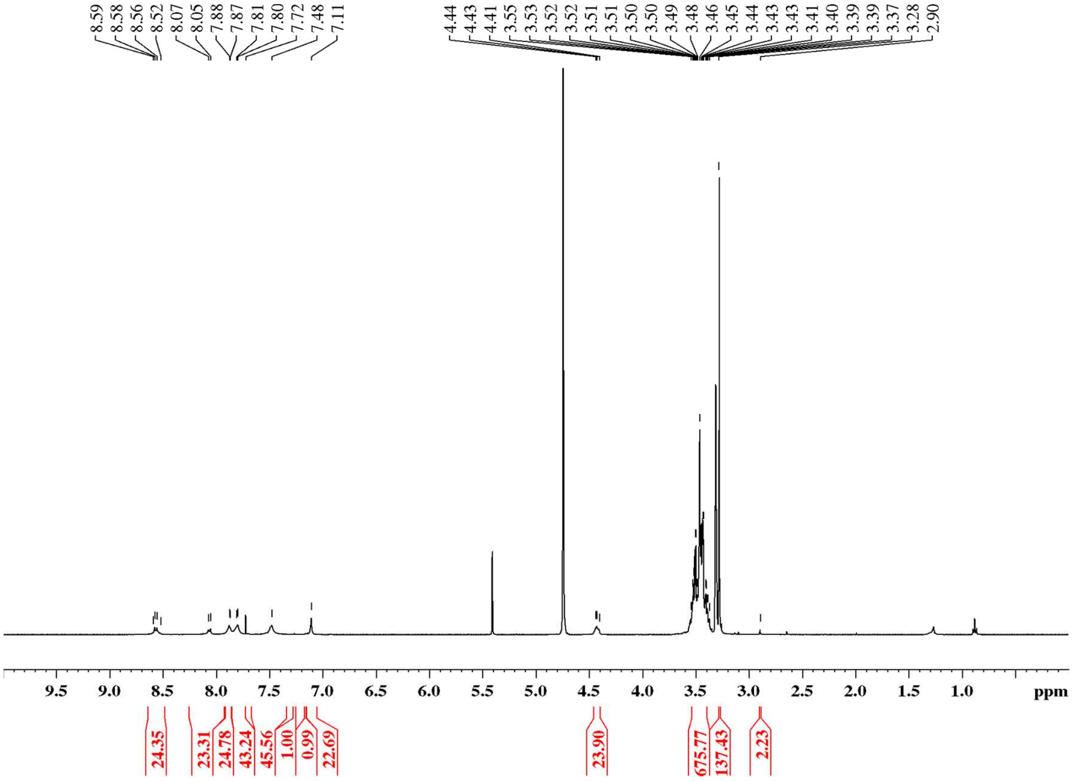

Figure S30. ^{1}H NMR spectrum (400 MHz, CD_3OD/CD_2Cl_2, *v:v*, 5:1, 295 K) of **4a**.

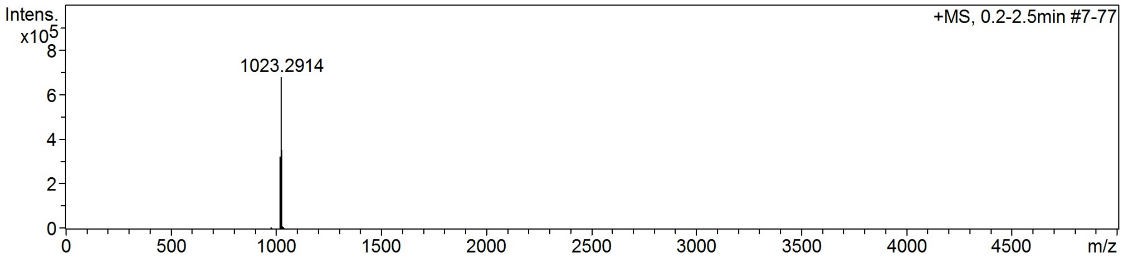

Figure S31. HR-MS spectrum (ESI-TOF, $CHCl_3$/MeCN) of **S3.**

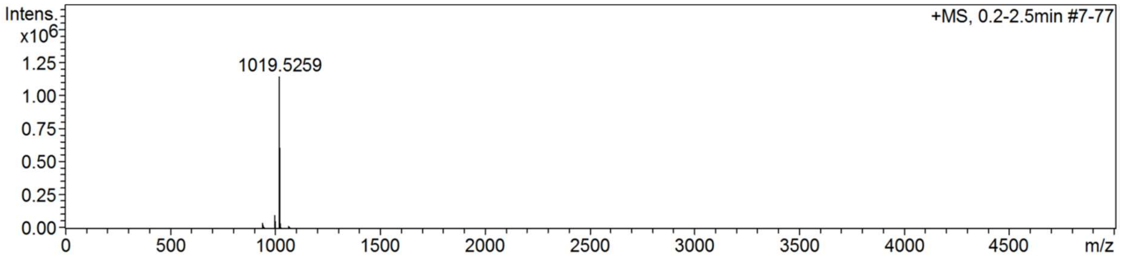

Figure S32. HR-MS spectrum (ESI-TOF, $CHCl_3$/MeCN) of **1.**

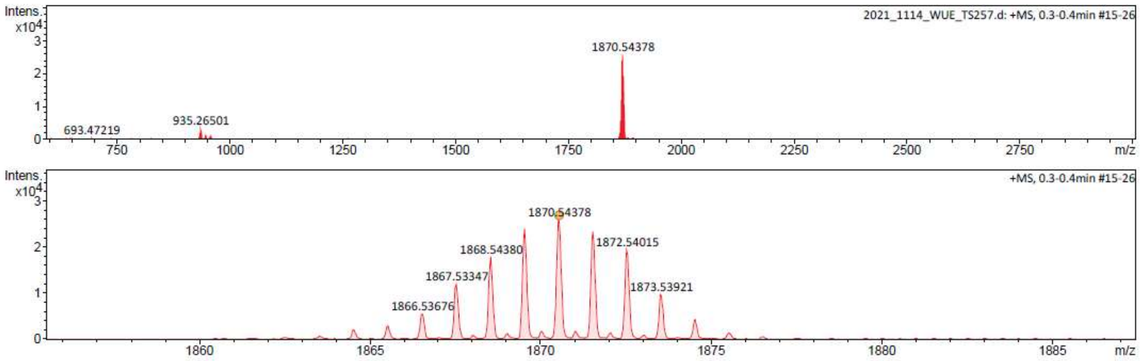

Figure S33. HR-MS spectrum (ESI-TOF, $CHCl_3$/MeCN) of **4c.**

Literature

[S1] C. Baskar, Y.-H. Lai, S. Valiyaveettil, *Macromolecules* **2001**, *34*, 6255–6260.

[S2] Y. Kim, J. choi, C. Lee, Y. Kim, C. Kim, T. L. Nguyen, B. Gautam, K. Gundogdu, H. Y. woo, B. J. Kim, *Chem.* **2018**, *30*, 5663–5672.

[S3] T. Song, X. Rao, Y. Cui, Y. Yang, G. Qian, *J. Alloys Compd.* **2013**, *555*, 22–27.

[S4] F. Li, B. Zhang, X. Li, Y. Jiang, L. Chen, Y. Li, L. Sun, *Angew. Chem. Int. Ed* **2011**, *50*, 12276–12279.

[S5] B. D. Sherman, Y. Xie, M. V. Sheridan, D. Wang, D. W. Shaffer, T. J. Meyer, J. J. Concepcion, *ACS Energy Lett.* **2017**, *2*, 124–128.

[S6] E. P. Friis, J. E. T. Andersen, L. L. Madsen, N. Bonander, P. Møller, J. Ulstrup, *Electrochim. Acta* **1998**, *43*, 1114–1122.

[S7] X. Guo, E. Laryea, M. Wilhelm, B. Luy, H. Nirschl, G. Guthausen, *Macromol. Chem. Phys.* **2017**, *218*, 1600440.

[S8] N. H. Williamson, M. Nydén, M. Röding, *J. Magn. Reson.* **2016**, *267*, 54–62.

[S9] BIOVIA, Materials Studio 2017, Release 17.1.0.48, San Diego: BIOVIA, **2007**.

[S10] B.-M. Cheng, M. Bahou, W.-C. Chen, C. Yui, Y.-P. Lee, L. C. Lee, *J. Chem. Phys.* **2022**, *117*, 1633–1640.

[S11] X. Sala, S. Maji, R. Bofill, J. García-Antón, L. Escriche, A. Llobet, *Acc. Chem. Res.* **2014**, *47*, 504–516.

[S12] F. Li, Y. Jiang, B. Zhang, F. Huang, Y. Gao, L. Sun, *Angew. Chem. Int. Ed.* **2012**, *51*, 2417-2420.

[S13] L. L. Zhang, Y. Gao, Z. Liu, X. Ding, Z. Yu, L. C. Sun, *Dalton Trans.* **2016**, *45*, 3814-3819.

[S14] S. Berardi, L. Francàs, S. Neudeck, S. Maji, J. Benet-Buchholz, F. Meyer, A. Llobet, *ChemSusChem* **2015**, *8*, 3688–3696.

[S15] M. Schulze, V. Kunz, P. D. Frischmann, F. Würthner, *Nat. Chem.* **2016**, *8*, 576-583.

[S16] A.-L. Meza-Chincha, J. O. Lindner, D. Schindler, D. Schmidt, A.-M. Krause, M. I. S. Röhr, R. Mitrić, F. Würthner, *Chem. Sci.* **2020**, *11*, 7654–7664.

[S17] A.-L. Meza-Chincha, D. Schindler, M. Natali, F. Würthner, *ChemPhotoChem* **2021**, *5*, 173–183.

[S18] D. Schindler, A. L. Meza-Chincha, M. Roth, F. Würthner, *Chem. Eur. J* **2021**, *27*, 16938–16946.

[S19] N. Noll, F. Würthner, *Chem. Eur. J* **2021**, *27*, 444–450.
